# Brain gene expression reveals pathways underlying nocturnal migratory restlessness

**DOI:** 10.1038/s41598-024-73033-3

**Published:** 2024-09-28

**Authors:** Valeria Marasco, Leonida Fusani, Patricia Haubensak, Gianni Pola, Steve Smith

**Affiliations:** 1 Department of Interdisciplinary Life Sciences, Research Institute of Wildlife Ecology, University of Veterinary Medicine Vienna, Savoyenstraße 1a, Vienna, 1160 Austria; 2 Department of Interdisciplinary Life Sciences, Konrad Lorenz Institute of Ethology, University of Veterinary Medicine Vienna, Savoyenstraße 1a, Vienna, A-1160 Austria; 3 Department of Behavioural and Cognitive Biology, University of Vienna, Djerassiplatz 1, Vienna, 1030 Austria; 4 Istituto Sperimentale Zootecnico per la Sicilia, via Roccazzo 85, 90135 Palermo, Italy

**Keywords:** Brain transcriptomics, Seasonality, Seasonal energy turnovers, Avian migration, Nocturnal restlessness, Lipid metabolism, Apolipoproteins, Animal behaviour, Animal physiology, High-throughput screening, Microarrays

## Abstract

Migration is one of the most extreme and energy demanding life history strategies to have evolved in the animal kingdom. In birds, champions of long-distance migrations, the seasonal emergence of the migratory phenotype is characterised by rapid physiological and metabolic remodelling, including substantial accumulation of fat stores and increases in nocturnality. The molecular underpinnings and brain adaptations to seasonal migrations remain poorly understood. Here, we exposed Common quails (*Coturnix coturnix*) to controlled changes in day length to simulate southward autumn migration, and then blocked the photoperiod until birds entered the non-migratory wintering phase. We first performed *de novo* RNA-Sequencing from selected brain samples (hypothalamus) collected from birds at a standardised time at night, either in a migratory state (when restlessness was highest and at their body mass peak), or in a non-migratory state and conducted differential gene expression and functional pathways analyses. We found that the migratory state was associated with up-regulation of a few, yet functionally well defined, gene expression networks implicated in fat trafficking, protein and carbohydrate metabolism. Further analyses that focused on candidate genes (apolipoprotein H or APOH, lysosomal associated membrane protein-2 or LAMP2) from samples collected during the day or night across the entire study population suggested differences in the expression of these genes depending on the time of the day with the largest expression levels being found in the migratory birds sampled at night. We also found that expression of APOH was positively associated with levels of nocturnal activity in the migratory birds; such an association was absent within the non-migratory birds. Our results provide novel experimental evidence revealing that hypothalamic changes in expression of apolipoprotein pathways, which regulate the circulatory transport of lipids, are likely key regulatory activators of nocturnal migratory movements. Our study paves the way for performing deeper functional investigations on seasonal molecular remodelling underlying the development of the migratory phenotype.

## Introduction

The long-distance migrations performed by billions of birds every year are considered one of the most remarkable examples of phenotypic flexibility and adaptations to seasonal fluctuations in resource availability. The seasonal development of the migratory phenotype involves rapid behavioural, physiological and metabolic remodelling, including hyperphagia, large accumulation of fat stores (up to 50% of body mass), and increases in muscle mass^1–4^. Fat is the main source of energy required to support long-distance migratory flights in birds^1,5^. In addition, most birds undergo rapid switches in their activity rhythms as they perform endurance migratory flights at night despite being diurnal outside the migratory periods^3,6^. This increased level of nocturnality is commonly termed “*Zugunruhe*” when displayed by birds in captivity and this behaviour is considered a good proxy for the natural disposition to migrate^7–9^. Despite it being relatively well known that the timing of development of the migratory phenotype is activated by changes in daily photoperiod as well as endogenous programs^10,11^, the molecular underpinnings that allow these signals to change seasonally and daily remain largely unknown. Filling this knowledge gap is critical to understand how seasonality has driven the evolution of extreme physiological life-history strategies, such as migration and hibernation, enabling species to appropriately respond to fluctuating selection pressures on survival and reproduction^12^.

Quantitative genetic studies using pioneering selective breeding experiments in the migratory blackcap (*Sylvia atricapilla*), or others comparing related species or populations of the same species with distinct migratory strategies, demonstrate that the disposition to migrate, direction and distance of migration have, at least in part, an inherited genetic base^13–17^. While more recent research efforts have aimed to integrate genotyping of wild migratory birds with isotope analyses or advanced tracking techniques^18–20^, to date there are still no genes or loci that have consistently been shown to influence variation in migratory life-history traits across different species and, therefore, that could be used to identify a common genetic basis of seasonal migration. Thus, the theory that potentially all birds possess the required genetic machinery to migrate and that the disposition to migrate develops when a so called “liability variable” (e.g. the expression of a certain molecule/s) reaches critical thresholds remains a plausible one (“*threshold model of migration*” - Pulido, et al^21^.). The circannual and circadian dynamics of the migratory phenotype likely depends on gene by environment interactions through the integrated actions of various functional pathways^3^that could be experimentally unveiled by using powerful and tissue-specific high-throughput analyses, such as transcriptomics^15^.

The brain, primarily the hypothalamus, integrates behaviours related to the homeostatic regulation of body mass and locomotory signals in vertebrate taxa. Thus, the hypothalamus is thought to have a central role in the integration of neurophysiological signals mediating migration-related behaviours^22,23^. Relatively few studies to date have examined brain-derived global gene expression signatures in migratory birds across different seasonal states within individuals of either the same species [Northern Wheatear (*Oenanthe Oenanthe*)^24^; Swainson’s thrush (*Catharus ustulatus*)^25^; Black-headed bunting (*Emberiza melanocephala*)^26^), or subspecies [Willow warbler (*Phylloscopus trochilus t.* and *Phylloscopus t. ]acredula*)^27^). Broadly, these studies provide good evidence that processes involved in neuronal tissue development, ATP production, fatty acids protein binding, and lipid transport are generally enriched in association with the development of the migratory phenotype. Results from these studies also suggest that variation in the migratory state relies on relatively small changes in brain transcriptional regulation, with the largest differences reported by Johnston and colleague^25^with 188 differentially regulated genes in the Swainson´s thrush. Frias-Soler, et al^24^. performed in-depth analyses to compare the wheatears´ brain transcriptome data with results from the Swainson´s thrush study^25^and the Willow warbler study^27^and found no shared patterns of gene expression among these three migratory species. These results could be due to several study factors including nomenclature differences in gene annotation among studies^24^, brain tissue selection (whole brain vs. hypothalamus) given that brain transcriptome profiles are strongly region/area-dependent^28^, photoperiod differences at the time of tissue sampling^26^, and limited control for the potential effect of sex- and age-specific differences in gene expression patterns^29,30^. Thus, it remains unclear to what extent the expression of the migratory phenotype is controlled by the action of common regulatory network neuronal pathways.

In this study, we exposed captive young adult Common quails (*Coturnix coturnix*) to controlled changes in day length to simulate autumn migration followed by a non-migratory wintering phase. This Galliform species shows very similar physiological adaptations to those found in most migratory Passerines, including rapid and substantial increases in subcutaneous fat stores and amount of nocturnality upon the onset of the migratory seasons^31–33^. Their high relatedness to *Gallus gallus*, by far the best characterised and annotated genome in birds, in combination with the good background on their photoperiodism and seasonality^34^, makes the migratory Common quail an ideal, yet non-traditional study system, for examining regulatory genome-wide scale expression changes while carefully controlling for their seasonal/migratory state. We thus first performed an RNA-Sequencing experiment to compare hypothalamic transcriptome profiles between birds sampled during the migratory phase (i.e. when they reached their peak in body mass / fat stores and displayed migratory restlessness) and birds sampled during the non-migratory phase (i.e. when they returned to their physiological baselines). In both sampling phases, the brain hypothalamic samples selected for the RNA-Sequencing experiment were all collected at a standardised time of the night and were characterised by the most divergent phenotypes on levels of nocturnality and fattening. In order to evaluate robustness of the RNA-Sequencing data and to assess the extent to which results could be generalised to the entire study population, we performed follow-up analyses to determine whether the predicted target genes were also influenced by the time of the day in which the birds were sampled (day vs. night hours) and to assess if within-individual variation in gene expression levels of the target genes predicted robust proxies of the migratory state.

## Materials and methods

This study was performed on experimental subjects used for a larger experiment (full details on experimental design in^35^). Briefly, eggs of Common quail were obtained from our breeding colony (Konrad Lorenz Institute of Ethology, Vetmeduni Vienna, Austria), which derived from wild founders with no admixture with the domestic Japanese quail^36^. The eggs were artificially incubated (incubator: MG 70/100 F, FIEM srl, Italia) and hatchlings reared under a 16:8 h light: dark until they were 7 weeks of age. When they could be sexed (4 weeks of age), the birds were housed in sex-mixed groups of 11–12 per enclosure (6 enclosures in total). Food (turkey starter, Lagerhaus, Austria) and water were always provided *ad libitum*. All birds were housed within a single room at controlled ambient temperature (20–24 °C).

The experiment was performed in compliance with the Austria legislation with approval of the Ethics Committee of the University of Veterinary Medicine Vienna, and the Federal Ministry of Science, Research and Economy (BMWFW-68.205/0037-WF/V/3b/2017). This study is reported in accordance with the ARRIVE guidelines.

### Photoperiod manipulation and assessment of physiological migratory state

**Fig. 1:**
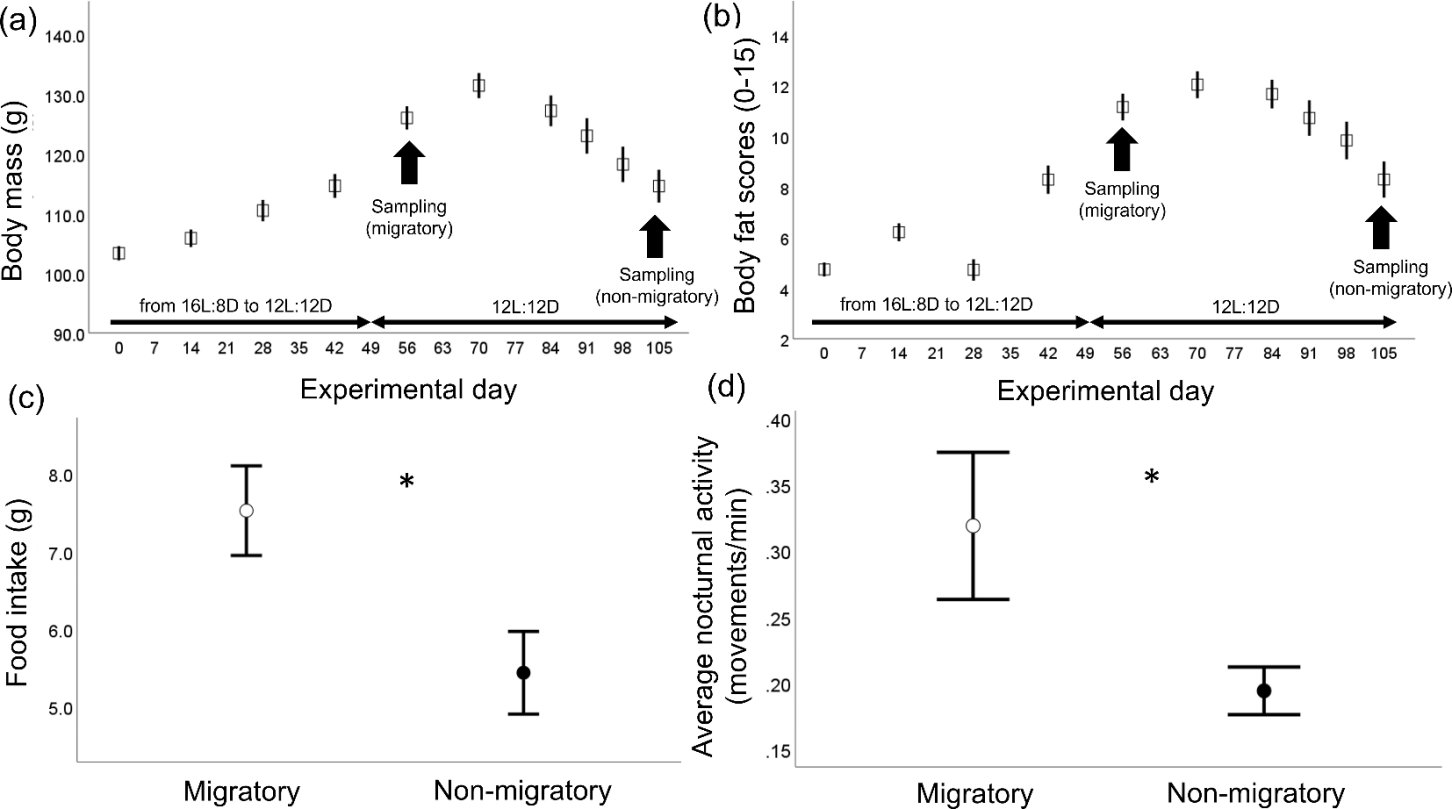
Physiological assessment of phenotypic state in the study population of Common quail (Coturnix coturnix); the experiment began in 7 weeks old birds (n = 68 in total) raised under long photoperiod (16:8 h L:D cycle). (a) Changes in body mass and (b) total subcutaneous fat scores (assessed in the furcular, scapular and abdomen following^31^) over the experimental period; the horizontal arrows in figures (**a**) and (**b**) indicate the weekly reduction in daylight (experimental days 0-49) after which the photoperiod was kept constant until termination of the experiment; sample size from experimental days 0-56: 68 birds, sample size from experimental days 70-105: 35 birds. The vertical arrows in figures (**a**) and (**b**) indicate the start of sampling procedures when the birds were singly housed for measurements of daily food intake (**c**) and nocturnal activity levels (**d**) 2-3 days prior tissue sampling separately by sampling phase (migratory: experimental days 56-70; non-migratory: experimental days 105-119). Data are presented as means ± se; * indicates *p-value* < 0.05. Figures previously published in Marasco et al.^33^ with minor modifications.

At seven weeks of age (indicated as day 0), all study birds (female = 34, male = 34, total *n*= 68) were exposed to a gradual reduction of day length (30 min/week) until the photoperiod reached 12:12 h light (L):dark (D) (i.e. experimental day 49), after which it was maintained under this regime until the end of the experiment. This light:dark schedule simulated autumn migration to (sub)tropical regions followed by a sedentary stage at the wintering grounds. As reported elsewhere^32,33^, quails increased their body mass (on average 22%) through accumulation of body fat as the days were shortening (experimental days 0–49) reaching the peak of fattening upon experimental days 56-70, while the birds rapidly lost body mass (on average 13%) due to depletion of fat scores in the experimental phase in which the photoperiod was kept constant (experimental days 70–105) – see Fig. 1a-b, and Table S1 in Supplementary Material. To further evaluate the physiological state of the experimental birds, the birds at each enclosure were simultaneously moved into single cages (100 × 100 × 100 cm) two/three days prior to tissue sampling for individual measurements of food intake and nocturnal activity levels (see Supplementary Material 2 for full details on Methodology), which are ecologically relevant proxies of migratory state in birds kept in the laboratory. As previously reported^33^, migratory birds also ate more and were more active at night compared to the non-migratory birds (see Fig. 1c-d and Table S2 in Supplementary Material 2). Therefore, the photoperiod manipulation triggered marked physiological shifts corresponding to the activation and de-activation of the migratory state mimicking seasonal timing in the wild.

## Sampling timeline and brain collection

As reported elsewhere^33^, at experimental day 56 (i.e. corresponding to the peak of fattening, see Fig. 1a), 33 out of 68 birds (all birds within three enclosures) were randomly chosen and allocated to the migratory sampling phase (experimental days 56–70); of these, 16 birds [7 female and 9 male] were sampled at 9:00 h (three hours after lights on), and 17 [8 female and 9 male] were sampled at 21:00 h (three hours after lights off). The remaining birds in the other three enclosures were allocated to the non-migratory sampling phase (experimental days 105–119); of this, 18 [9 female and 9 male] were sampled at 9:00 h and 17 [10 female and 7 male] were sampled at 21:00 h. We sampled up to two birds at the same sampling time point to collect tissues as close to the defined collection time as possible; thus, within each sampling phase, tissues were taken over 12 consecutive days. The night sampling was performed using a head lamp with red light^37^. Birds were weighed using an electronic balance to the nearest 0.01 g and immediately euthanized using an overdose of sodium pentobarbital (200 mg/ml) via IP injection. The brains were quickly dissected (within 6 min *post-mortem*), placed on dry ice and stored at − 80 °C until further dissections. Post-mortem visual inspection of gonadal size ascertained that none of the birds had a fully developed ovary or testes in agreement with the complete absence of foam production in the males and the reduced length of the cloacal vent in both sexes (both proxies of sexual development in quails^38^) assessed prior tissue sampling (experimental day 56, female: 1.2 ± 0.1, male: 2.4 ± 0.2; experimental day 105, female: 0.9 ± 0.03, male: 2.5 ± 0.2; all measurements were taken using a digital calliper to the nearest 0.1 mm), indicating that the birds were not physiologically ready to reproduce^39^.

Punch dissections to isolate the hypothalamus were performed as previously described^40^. Briefly, the brains were placed ventral side up into a frozen brain holder matrix (Hugo Sachs Elektronik, Germany) with a 1 mm graduated scale. Two blades were positioned ∼ 4 mm from the rostral pole and ∼2 from the cerebellum to obtain a 2 mm-thick coronal section. The cutting plane was adjusted to match as closely as possible the plane of the chicken brain atlas^41^ (coronal brain sections interaural 2.08–2.56 mm). One single punch (diameter: 2 mm) per brain was obtained from the basal hypothalamus spanning the third ventricle, and immediately stored at − 80 °C until RNA isolation.

### Total RNA isolation

Total RNA was extracted using the Rneasy Microarray Tissue Mini Kit (Qiagen, Hilden, Germany). RNA purity and integrity were assessed using a Nanodrop spectrophometer (ThermScientific, Waltham, MA, USA) and Agilent 2100 Bioanalyzer (Agilent Technologies, Santa Clara, CA, USA), respectively. Hypothalamic RNA concentrations averaged 86.30 ± 5.80 ng/µl, ratio 260/280: 2.01 ± 0.01, Agilent RNA integrity number (RIN) scores averaged 9.03 ± 0.06 (mean ± se for all).

### RNA-sequencing workflow

#### Selection of birds for RNA-sequencing

Out of 34 birds sampled at night in both sampling phases, 23 were selected for RNA-Sequencing analysis (migratory: *n* = 11 [6 females and 5 males]; non-migratory: *n* = 12 [7 females and 5 males]). The selection was based on the nocturnal locomotor activity levels and the body mass at sampling by choosing the birds that showed the largest differences in these two proxies of migratory state (Fig. S1, Supplementary Material 2) in order to maximise statistical power. There was a high within-individual measurement repeatability in the average nocturnal activity levels over the two consecutive nights prior the brain sampling (*r* = 0.9, *p*< 0.0001, assessed following^42^). We thus based the selection of birds for the RNA-Sequencing processing and further statistics on the nocturnal activity data from the first night of single housing; we focused on average nocturnal activity levels during the first part of the night (18:00 h-00:00 h) as this is considered to be a more biologically relevant proxy of nocturnal restlessness^43,44^. However, results do not change qualitatively when considering average nocturnal activity levels during the entire night as average activity values during the first part of the night and the entire night were highly correlated (Pearson´s *r* ≥ 0.95, *p* < 0.001 for both nights of single housing).

### cDNA library preparation and sequencing strategies

We used two different RNA-sequencing strategies. To obtain a *de novo assembly* for our study species, a barcoded library was prepared from each sample using Lexogen Sense mRNA- sequencing Library Preparation kit (Lexogen, Vienna, Austria) according to the manufacturer’s instructions. The prepared cDNA libraries were then sequenced paired-end in one lane with a sequencing run length of 125 on a HiSeq V4 platform at the Vienna Biocenter (Vienna, Austria). To obtain sufficient read depth for transcript quantification, each cDNA library was additionally sequenced on a HiSeq V4 platform using 50 nt single-end sequencing at the Vienna Biocenter (Vienna, Austria). This hybrid sequencing approach was chosen to provide optimal *de novo* transcriptome assembly read length data while also providing enough read depth to approximate differential expression levels.

### Transcriptome assembly and annotation

The detailed pipeline used for compiling the reference genome has been described in full detail elsewhere^40^. Briefly, the *de novo*assembly was obtained using the Trinity software (v2.7.0) and it was comprehensively annotated using FunctionAnnotator^45^, Trinotate v3.2.0^46^ and eggNOG^47^. Our high-quality final transcriptome consisted of 22,293 transcripts of which 21,551 (97%) were complemented with annotation data mostly relying on *Gallus gallus* (see Supplementary Material 1).

### Transcript quantification and differential gene expression analysis

Both 50 nt single-end and 125 nt paired-end raw reads were pre-processed following standard guidelines^48^. After conversion to fastq format, we used the software Trimmomatic v0.38^49^ to remove Illumina adapter sequences (up to two mismatches were allowed when recognizing adaptor sequences) and to filter out low quality reads (average Phred quality score < 30 in a sliding window of 8 base pairs across the entire reads and reads below a minimum length of 50). The quality filtered single-end and paired-end reads were then merged together to increase sequencing depth. We then quantified abundances of transcripts at the gene level using the ultra-fast alignment-free method Kallisto that examines k-mer abundances in the reads and in the resulting assemblies^50^. The resulting count matrix containing all the biological samples was then assessed for differential gene expression using DESeq2 based on Generalized Linear models using the negative binomial distribution^51^. As our main aim was to determine the overall differences in gene expression patterns associated with sampling phase (migratory vs. non-migratory) while controlling for the presence of the two sexes, we first performed an additive model with sampling time and sex as fixed effect terms. To explore the extent to which fold changes attributable to the sampling phase were sex-specific due to potential interactive effects, we performed an additional model by combining sampling phase and sex into a single factor to determine the effect of sampling phase for male, or for female following recommendations from the software authors (DESeq2 vignette, https://bioconductor.org/packages/devel/bioc/vignettes/DESeq2/inst/doc/DESeq2.html). Differential expression was defined as a *p-value*≤ 0.1 after conservative FDR correction using Benjamini and Hochberg^52^.

### Functional analyses

We used the software Ingenuity Pathway Analysis (IPA; Qiagen, Hilden, Germany) to identify over-represented biological pathways (i.e. molecular, cellular and physiological functions) and networks associated with the differentially expressed gene lists due to sampling phase, sex, or the interaction between these two factors (i.e. three core IPA analyses in total, default settings). In IPA, each gene was mapped to its corresponding gene object in the Ingenuity Knowledge Base (IKB) via ortholog mapping to their vertebrate counterparts (predominantly based on human, mouse, and rat). The IKB converted each gene list into a shorter dataset of non-redundant “focus” genes. These focus genes were then mapped to molecular networks in IKB on the basis of their inter-connectivity and ranked by scores. These scores indicate the probability that a collection of genes equal to or greater than the number in a network was a result of chance alone.

### Droplet digital PCR (ddPCR™)

At completion of the RNA-Sequencing analyses, we performed ddPCR to measure gene expression for two selected genes showing high fold change differences in relation to the overall effect of sampling phase (DESeq2: fold changes: 1.9–2.5, *Wald statistic* ≥ 4.3) and that were indicated as biologically relevant by the functional analyses: apolipoprotein H (APOH), and lysosomal associated membrane protein-2 (LAMP2).

Importantly, these analyses were performed in all study subjects (i.e. within the birds selected for the RNA-Sequencing experiment and in all the remaining birds [*n* = 68 in total]). Thus, the ddPCR data served not only as a technical validation of the gene expression data obtained by RNA-Sequencing but also as a biological validation. The latter allowed us to assess the extent to which the RNA-Sequencing results could be generalised to the entire study population, thus in the birds that were sampled either during the day, or the night, within each sampling phase. Details on primer design, primer sequences and protocol for the Droplet Digital PCR ddPCR™ analyses are reported in Supplementary Methods and Table S3 (see Supplementary Material 2). We expressed gene expression levels as the relative ratio of the concentration (copies/µl) of the target gene (APOH or LAMP2) over the concentration of the candidate reference gene, glyceraldehyde-3-phosphate dehydrogenase (GAPDH). GAPDH was highly and uniformly expressed in the hypothalamic samples between the two sampling phases as indicated by the RNA-Sequencing results.

### Statistical analyses of ddPCR-generated data

Statistical analyses were performed in R 4.2.2^53^ integrated in RStudio 2023.6.1.524^54^. We first performed separate General Linear Models (GLMs) to assess for differences in hypothalamic APOH or LAMP2 expression levels in relation to the sampling phase (migratory or non-migratory), sex (male or female), time of the day (day or night), along with all the two-way interactions. Non-significant interactions (*p*> 0.05) were sequentially dropped from the final models. Significant interaction terms were further explored via pairwise post-hoc contrasts (R package “emmeans”^55^). We also performed separate GLMs by sampling phase and time at sampling to assess for potential links between each gene with respect to levels of food intake and nocturnal activity. When appropriate, gene expression data were ln-transformed to improve normality of model residuals. All models met the assumptions of normality and homogeneity, which were assessed via graphical diagnostics of the residuals^56^. Unless otherwise specified, descriptive statistics are provided as means ± se.

## Results

### RNA-sequencing differential gene expression analyses

**Fig. 2:**
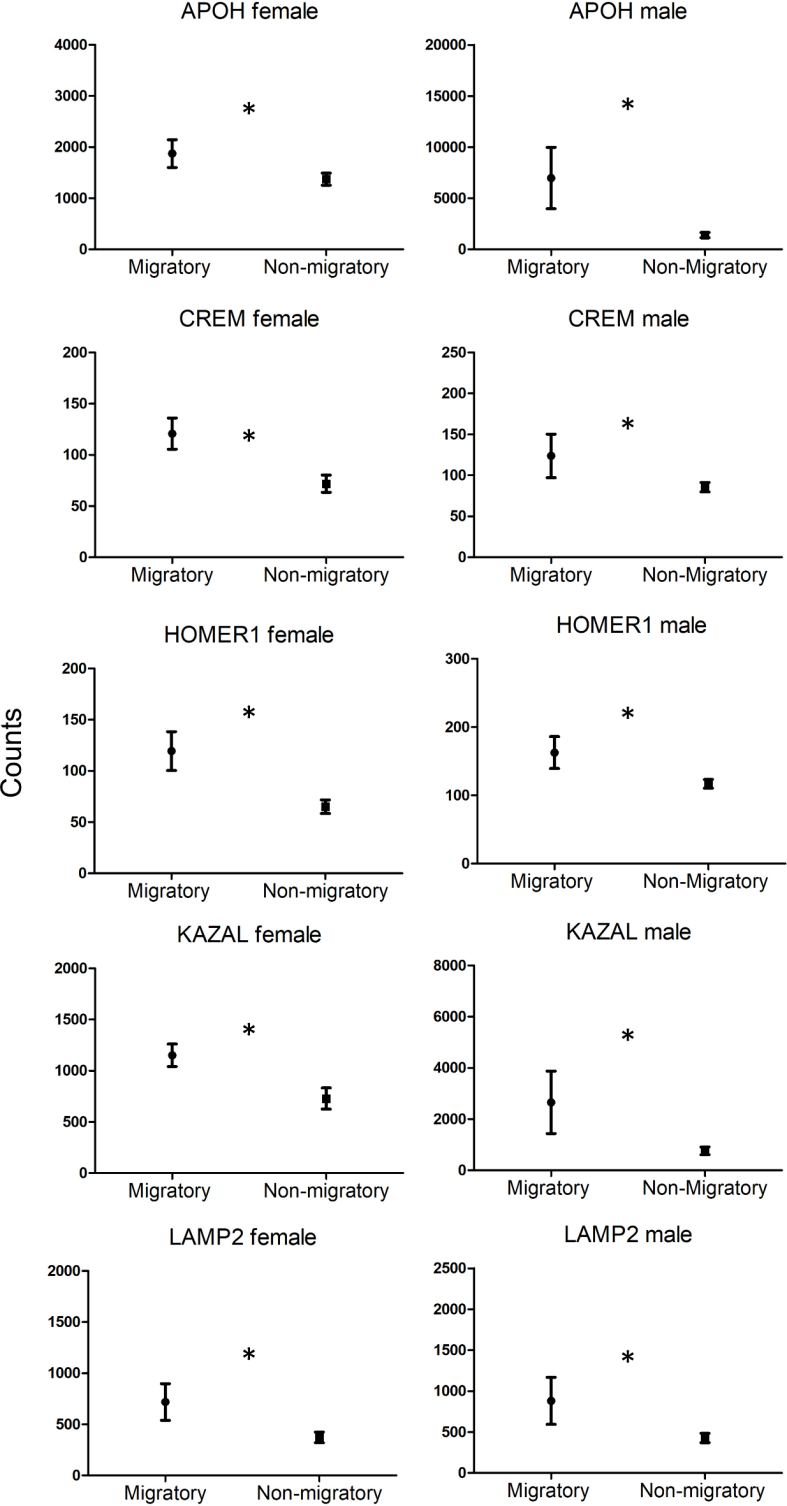
Normalised gene abundance estimate (transcripts per million, TPM; Kallisto software) of the top five up-regulated genes during the migratory phase compared to the non-migratory phase, separately by sex (see Table 1 for statistics). Data are shown as mean ± se; * indicates corrected *p-value: FDR* ≤ 0.1.

We found 16 genes differentially expressed due to the overall effect of sampling phase. Out of these 16 genes, 11 genes were up-regulated in the migratory birds compared to the non-migratory birds (the top 5 up-regulated genes are shown in Fig. 2). Two hundred twenty-five genes were differentially expressed between male and female quails (43 out of 225 genes were down-regulated in males compared to female birds). Additionally, ninety three genes were indicated as differentially expressed between the two sampling phases within the male quail (of which 78 genes were up-regulated during the migratory phase compared to the non-migratory phase) and 4 genes within the female quail (of which 3 genes were up-regulated during the migratory phase compared to the non-migratory phase). Of the 93 genes differentially expressed within the male samples, only 3 genes (APOH, IGE-HUMAN, and KAZAL) were also detected as differentially expressed due to the overall effect of sampling phase suggesting that the up-regulation of these genes during the migratory phase was of different intensity between the two sexes (see also Fig. 2). Out of the 4 genes differentially expressed within the female samples, 2 genes were also found differentially expressed due to the overall effect of sampling phase (CREM, HOMER1) possibly due to a stronger up-regulation of these genes during the migratory state in females compared to males (see Fig. 2). Full statistics of the top 10 up- or down-regulated significant transcripts with the lowest FDR-values from each factor are shown in Table 1a-c  (full statistics is provided in Supplementary Material 1).

### Functional pathways and network analyses

The differentially expressed genes due to the overall effect of sampling phase (16 genes), sex (225 genes), or the effect of sampling phase for the male quail were submitted to IPA software (i.e. three core IPA analyses in total). We only submitted the genes differentially expressed between the two sampling phases in the male samples (93 genes) due to the low number of genes (*n* = 4) differentially expressed in the female samples. The number of mapped non-redundant genes to the IKB in each of the three analyses were 13 (sampling phase), 200 (sex), and 82 (effect of sampling phase for the male). In Table 2(a-c), we reported the top 5 enriched molecular and physiological functions associated in each IPA core analyses.

**Table 1:** Top 10 up- or down-regulated differentially expressed genes (corrected p-value: FDR ≤ 0.1) due to (a) the overall effect of sampling phase (migratory or non-migratory), (b) the overall effect of sex (male or female), and (c) the effect of sampling phase for each sex. NA indicates un-characterised genes. In (a) we reported all genes with FDR ≤ 0.1 (n = 16). Log2 FC = log2 fold change; Stat = Wald statistic; p-value = Wald test p-value; FDR = corrected p-value.

**(a) Sampling phase**						
*Up-regulated genes under category “Migratory”*						
**Trinity_id**	**Gene name**	**Protein**	**Log2 FC**	**Stat**	**p-value**	**FDR**
TRINITY_DN2598_c0_g1	CREM	cAMP-responsive element modulator	0.82	5.43	< 0.0001	< 0.01
TRINITY_DN11216_c0_g1	APOH	Beta-2-glycoprotein 1	1.24	4.36	< 0.0001	0.04
TRINITY_DN46792_c0_g1	KAZAL	Serine protease inhibitor Kazal-type 5	1.18	4.34	< 0.0001	0.04
TRINITY_DN46896_c0_g1	HOMER1	Homer protein homolog 1	0.73	4.37	< 0.0001	0.04
TRINITY_DN4209_c0_g1	LAMP2	Lysosome-associated membrane glycoprotein 2	0.96	4.29	< 0.0001	0.04
TRINITY_DN15319_c0_g1	IGE_HUMAN	Immunoglobulin epsilon heavy chain	2.57	4.17	< 0.0001	0.05
TRINITY_DN5843_c0_g1	WNT4	Protein Wnt-4	1.17	4.12	< 0.0001	0.05
TRINITY_DN2821_c0_g1	etnppl	Ethanolamine-phosphate phospho-lyase	1.37	3.97	< 0.0001	0.07
TRINITY_DN17972_c1_g1	rpb-1	DNA-directed RNA polymerase II subunit RPB1	1.29	3.80	< 0.0001	0.10
TRINITY_DN8200_c2_g1	TBC1D5	TBC1 domain family member 5	1.13	3.79	< 0.0001	0.10
TRINITY_DN4786_c0_g1	RAB7B	Ras-related protein Rab-7b	1.00	3.80	< 0.0001	0.10
*Down-regulated genes under category “Migratory”*						
**Trinity_id**	**Gene name**	**Protein**	**Log2 FC**	**Stat**	**p-value**	**FDR**
TRINITY_DN11301_c0_g1	PCDHGA2	Protocadherin gamma-A2	-0.53	-4.12	< 0.0001	0.05
TRINITY_DN2249_c0_g1	SLC16A2	Monocarboxylate transporter 8	-0.76	-4.11	< 0.0001	0.05
TRINITY_DN3863_c0_g1	RCHY1	RING finger and CHY zinc finger domain-containing protein 1	-0.47	-3.99	< 0.0001	0.07
TRINITY_DN1950_c0_g3	ZN398	Zinc finger protein 398	-0.47	-3.93	< 0.0001	0.07
TRINITY_DN19371_c0_g1	CD048_CHICK	europeptide-like protein C4orf48 homolog	-0.62	-3.84	< 0.0001	0.10
**(b) Sex**						
*Up-regulated genes under category “Female”*						
**Trinity_id**	**Gene name**	**Protein**	**Log2 FC**	**Stat**	**p-value**	**FDR**
TRINITY_DN16909_c0_g1	ST8SIA3	Sia-alpha-2,3-Gal-beta-1,4-GlcNAc-R:alpha 2,8-sialyltransferase	9.24	10.56	< 0.0001	< 0.01
TRINITY_DN15660_c0_g1	Ubap2	Ubiquitin-associated protein 2	9.22	10.48	< 0.0001	< 0.01
TRINITY_DN3266_c0_g2	MIER3	Mesoderm induction early response protein 3	7.01	8.52	< 0.0001	< 0.01
TRINITY_DN4791_c0_g1	Ubap2	Ubiquitin-associated protein 2	6.59	8.41	< 0.0001	< 0.01
TRINITY_DN51862_c0_g1	ZFAND5	AN1-type zinc finger protein 5	0.98	5.73	< 0.0001	< 0.01
TRINITY_DN20767_c0_g1	POLR1B	DNA-directed RNA polymerase I subunit RPA2	0.78	4.21	< 0.0001	0.01
TRINITY_DN16025_c0_g2	NA	NA	1.26	4.15	< 0.0001	0.01
TRINITY_DN4614_c0_g1	NMRAL1	NmrA-like family domain-containing protein 1	0.78	3.85	< 0.0001	0.02
TRINITY_DN26960_c0_g1	Acot11	Acyl-coenzyme A thioesterase 11	0.67	3.83	< 0.0001	0.02
TRINITY_DN300_c0_g1	SPINZ	Spindlin-Z	0.79	3.80	< 0.0001	0.02
*Down-regulated genes under category “Female”*						
**Trinity_id**	**Gene name**	**Protein**	**Log2 FC**	**Stat**	**p-value**	**FDR**
TRINITY_DN8440_c0_g1	ATP5F1A	ATP synthase subunit alpha, mitochondrial	-0.92	-6.47	< 0.0001	< 0.01
TRINITY_DN14645_c0_g1	PLIN2	Perilipin-2	-0.98	-6.36	< 0.0001	< 0.01
TRINITY_DN96490_c0_g2	NIPSNAP3A	Protein NipSnap homolog 3A	-1.09	-5.84	< 0.0001	< 0.01
TRINITY_DN2659_c0_g2	TPGS2	Tubulin polyglutamylase complex subunit 2	-1.05	-5.90	< 0.0001	< 0.01
TRINITY_DN19867_c0_g1	WDR36	WD repeat-containing protein 36	-0.95	-5.89	< 0.0001	< 0.01
TRINITY_DN10863_c0_g2	RPS6	40S ribosomal protein S6	-0.92	-5.85	< 0.0001	< 0.01
TRINITY_DN22956_c0_g1	nol6	Nucleolar protein 6	-0.81	-5.85	< 0.0001	< 0.01
TRINITY_DN40324_c0_g1	ZFAND5	AN1-type zinc finger protein 5	-1.01	-5.79	< 0.0001	< 0.01
TRINITY_DN8222_c0_g1	MRPS30	28S ribosomal protein S30, mitochondrial	-0.92	-5.71	< 0.0001	< 0.01
TRINITY_DN15997_c0_g1	ELP1	Elongator complex protein 1	-0.85	-5.27	< 0.0001	< 0.01
**(c) Effect of sampling phase for each sex**						
*Up-regulated genes in males under category “Migratory”*						
**Trinity_id**	**Gene name**	**Protein**	**Log2 FC**	**Stat**	**p-value**	**FDR**
TRINITY_DN11216_c0_g1	APOH	beta-2-glycoprotein 1 precursor	2.17	5.94	< 0.0001	< 0.01
TRINITY_DN83656_c0_g1	HO-1	PREDICTED: heme oxygenase 1	1.68	4.96	< 0.0001	0.00
TRINITY_DN35380_c1_g1	AQP1	Aquaporin-1	2.41	4.81	< 0.0001	0.00
TRINITY_DN5501_c0_g1	Sgk2	serine/threonine-protein kinase Sgk2 isoform X13	0.39	4.70	< 0.0001	0.01
TRINITY_DN12809_c1_g1	FAR1	fatty acyl-CoA reductase 1 isoform X3	0.37	4.56	< 0.0001	0.01
TRINITY_DN1190_c0_g2	ENPP2	ectonucleotide pyrophosphatase	0.41	4.56	< 0.0001	0.01
TRINITY_DN5850_c0_g1	HYCCI_CHICK	hyccin	0.30	4.37	< 0.0001	0.02
TRINITY_DN46792_c0_g1	KAZAL	Kazal-type serine protease inhibitor domain-containing protein 1	0.39	4.32	< 0.0001	0.02
TRINITY_DN8748_c0_g2	EFHD1	EF-hand domain-containing protein D1	0.29	4.29	< 0.0001	0.02
TRINITY_DN15319_c0_g1	IGE_HUMAN	Immunoglobulin epsilon heavy chain	0.90	4.22	< 0.0001	0.02
*Down-regulated genes in males under category “Migratory”*						
**Trinity_id**	**Gene name**	**Protein**	**Log2 FC**	**Stat**	**p-value**	**FDR**
TRINITY_DN334_c0_g1	NA	NA	-27.11	-7.22	< 0.0001	< 0.01
TRINITY_DN21754_c0_g1	NA	NA	-1.86	-4.38	< 0.0001	0.02
TRINITY_DN2621_c0_g1	CHRNA1	Acetylcholine receptor subunit alpha	-1.94	-4.30	< 0.0001	0.02
TRINITY_DN8021_c0_g1	ALK	ALK tyrosine kinase receptor	-0.91	-4.22	< 0.0001	0.02
TRINITY_DN1108_c0_g1	PPIL6	Probable inactive peptidyl-prolyl cis-trans isomerase-like 6	-1.74	-4.00	< 0.0001	0.04
TRINITY_DN120_c6_g1	OBSCN	Obscurin	-2.34	-3.92	< 0.0001	0.05
TRINITY_DN4380_c0_g1	RTKN2	Rhotekin-2	-2.04	-3.91	< 0.0001	0.05
TRINITY_DN23403_c0_g2	C3orf20	Uncharacterised	-1.89	-3.74	< 0.0001	0.06
TRINITY_DN20197_c0_g1	AMYP_STRCA	Pancreatic alpha-amylase	-0.76	-3.71	< 0.0001	0.07
TRINITY_DN40734_c0_g1	NA	NA	-6.17	-3.61	< 0.0001	0.08
Up-regulated genes in females under category “Migratory”						
**Trinity_id**	**Gene name**	**Protein**	**Log2 FC**	**Stat**	**p-value**	**FDR**
TRINITY_DN2598_c0_g1	CREM	cAMP-responsive element modulator	0.93	4.54	< 0.0001	0.04
TRINITY_DN4227_c0_g1	NA	NA	3.65	4.33	< 0.0001	0.05
TRINITY_DN46896_c0_g1	HOMER1	Homer protein homolog 1	0.95	4.33	< 0.0001	0.05
Down-regulated genes in females under category “Migratory”						
**Trinity_id**	**Gene name**	**Protein**	**Log2 FC**	**Stat**	**p-value**	**FDR**
TRINITY_DN334_c0_g1	NA	NA	-22.24	-6.73	< 0.0001	0.00

**Fig. 3:**
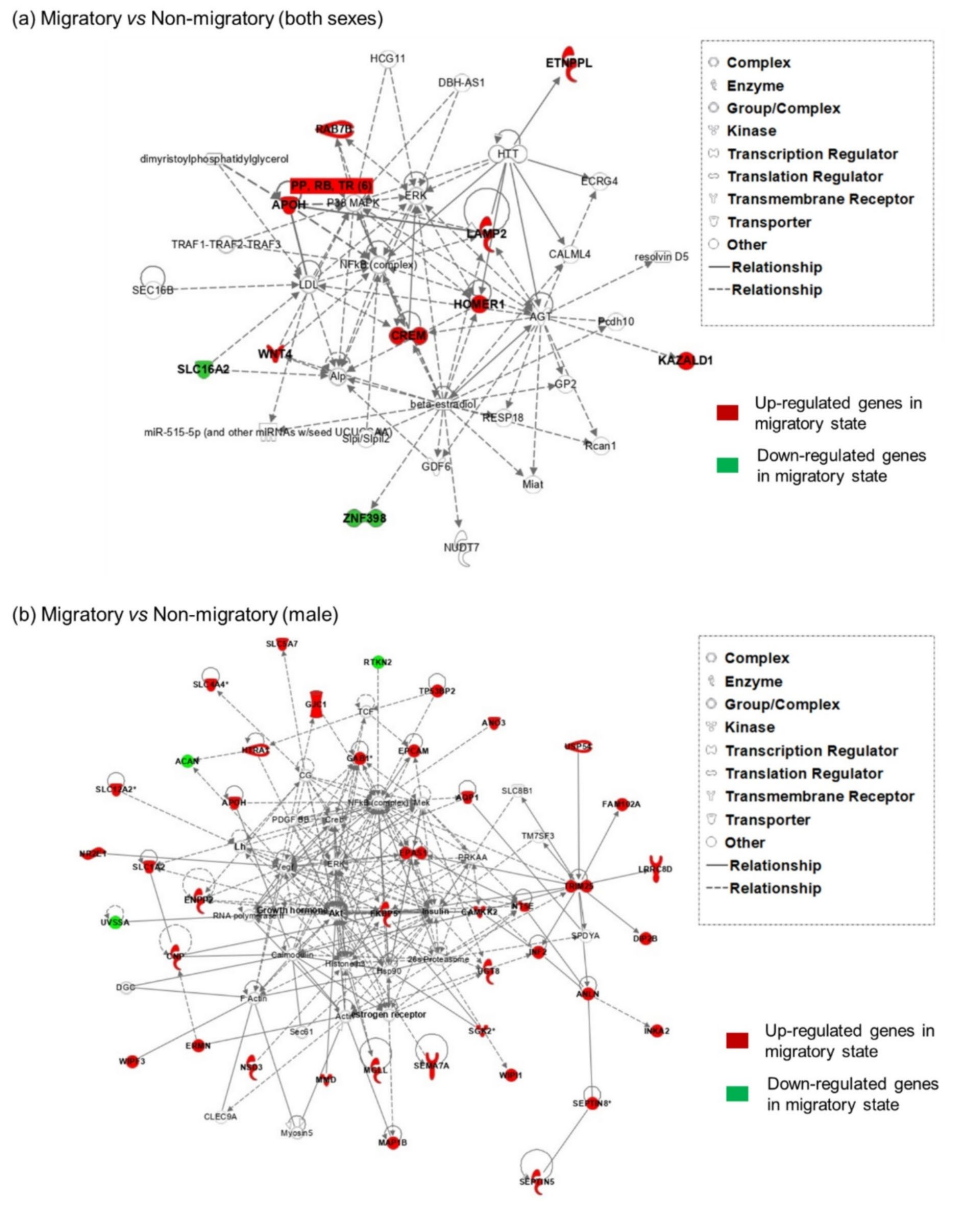
Top significant networks generated by Ingenuity Pathway Analysis (IPA) showing the down-regulated focus genes (green) and up-regulated focus genes (red) for the overall effect of sampling phase (migratory vs non-migratory) in (**a**) both sexes, or (**b**) only in the male birds. In (b) the top two significant networks were merged together (IPA default setting). Each network is displayed with nodes (i.e. genes) and edges (i.e. biological interactions among nodes); in white, the not user-specific molecules added into the network as a result of interactions with the submitted (i.e. user-specific) genes (green or red). Solid lines connecting distinct molecules indicate direct interactions between the nodes and dashed lines implied indirect interactions.

The network analysis of the genes associated with the overall effect of sampling phase revealed the existence of 2 significant networks (scores: 28 and 7, respectively) involving 10 and 3 of the focus genes, respectively (Table 3a). The top network incorporated all the focus genes except PCDHGA5, RCHY1, and TBC1D5 (Fig. 3a). The top five up-regulated genes in the migratory birds incorporated in the top network (APOH, CREM, HOMER1, KAZAL, and LAMP2) were associated with regulation of metabolism, accumulation of lipid, endocrine system development, and behaviour (see also Table 2a).

**Table 2:** Top five enriched biological processes as determined with IPA for the differentially expressed genes for (a) the overall effect of sampling phase, (b) sex, or (c) the effect of sampling phase for male quail.    .

(a) Sampling phase		
Molecular and Cellular Functions	p-value range	Molecules
Lipid metabolism	0.043-<0.0001	APOH, CREM, LAMP2, WNT4, ETNPPL, SLC16A2
Molecular Transport	0.044-<0.0001	APOH, CREM, LAMP2, WNT4, ETNPPL, SLC16A2, HOMER1
Small Molecule Biochemistry	0.048-<0.0001	APOH, CREM, LAMP2, WNT4, ETNPPL, SLC16A2, HOMER1
Vitamin and Mineral Metabolism	0.034-0.0002	CREM, WNT4, HOMER1
Amino Acid Metabolism	0.042-0.0002	LAMP2, SLC16A2, HOMER1

Physiological System Development and Functions	p-value range	Molecules
Endocrine System Development and Functions	0.048-0.0003	CREM, WNT4, SLC16A2
Behaviour	0.049-0.0006	HOMER1, CREM, SLC16A2
Connective Tissue Development and Function	0.058-0.0006	LAMP2, WNT4, SLC16A2
Embryonic Development	0.045-0.0006	CREM, WNT4, SLC16A2
Hematological System Development and Function	0.043-0.0006	CREM, WNT4, APOH

(b) Sex		
Molecular and Cellular Functions	p-value range	Molecules
Cellular Assembly and Organization	0.0085-<0.0001	ABCA1, AGTPBP1, AP3B1, AP3S1, APC, ARHGEF28, C9orf71, CATIP, CFK5R2, CEP120, CERT1, CLTA, CPLANE1, DAPK1, DLG1, DNAJA1, DPYSL3, DYM, EPG5, ERBIN, FER, IL6ST, LPAR1, MAP3K1, NPHS1, NTRK2, NUP155, DPGFA, PELO, PIAS2, PLAA, PPARGC1A, PRKAA1, PTCH1, PTPRA, Ptprd, PTPRK, RASA1, RICTOR, RPS6, SEMA4D, SLC25A46, SMARCA2, SPTLC1, TMEM38B, TMEM4AB, TRAK1, UBQLN1, UGT8, WDR36, ZNG462, ZSWIM6
Post-Translational Modification	0.008-<0.0001	ABCA1, ATG10, C9orf71, CCNHm, CDK5R2, CDK7, DAPK1, DGKQ, ERCC8, FER, FNTA, GCK, HMGCR, MAP3K1, NDUFS4, NIM1K, NTRK2, PDE4D, PDGFA, PIGG, PIK3C3, PPARGC1A, PRKAA1, PTPRA, RASA1, RICTOR, RNF20, SEMA4D, STSIA3, ST8SIA5, TOPORS, TRIM23
Lipid metabolism	0.009-<0.0001	ABCA1, ACAA2, ACER2, ACOT11, AP3B1, APC, APOD, ARMC5, CERT1, DGKQ, FAR2, GCK, GNE, HMGCR, HSD17B4, LPAR1, MAP3K1, MRAP, NPHS1, NTRK2, PDE8B, PDGFA, PIGG, PIK3C3, PLAA, PLIN2, PLPP1, PPARGC1A, PRKAA1, PTCH1, SLC16A2, SLC125A46, SLC44A1, SNCAIP, SPTLC1, ST8SIA3, ST8SIA5, TMEM38B, UGT8, XPA
Small Molecule Biochemistry	0.009-<0.0001	ABCA1, ACAA2, ACER2, ACOT11, AGTPBP1, AP3B1, APC, APOD, ARMC5, CENPN, CERT1, CMPK2, DGKQ, DIMT1, DLG1, ERCC8, FAR2, FER, GCK, GLIS3, GNE, HMGCR, HSD17B4, IL6ST, LPAR1, MAP3K1, MRAP, NANS, NDUFS4, NPHS1, NTRK2, Oasl2, Pde4d, PDE8B, PDGFA, PFAS, PIGG, PIK3C3, PLAA, PLIN2, PLPP1, PPARGC1A, PRKAA1, PTCH1, RASA1, RICTOR, RMI1, RNLS, RPS6, SEMA4D, SLC16A2, SLC25A46, SLC44A1, SNCAIP, SPTLC1, ST8SIA3, ST8SIA5, STARD4, TMEM38B, UGT8, XPA
Cellular Function and Maintenance	0.009-<0.0001	ABCA1, ACER2, ACOT11, AGTPBP1, AP3B1, AP3S1, APC, APOD, ARHGEF28, ARMC5, ATG10, C9orf72, CATIP, CDK5R2, CEP120, CERT1, CPLANE1, DAPK1, DLG1, DNAJA1, DPYSL3, DYM, ELP1, EPG5, ERBIN, FER, GCK, HOMER1, IL6ST, LIPT2, LMBR1L, LPAR1, MAP3K1, NDUFS4, NPHS1, NTRK2, PDGFA, PIAS2, PIK3C3, PJA2, PLAA, PPARGC1A, PRKAA1, PTCH1, PTPRA, Ptprd, PTPRK, RASA1, RICTOR, RMI1, RPS6, SEMA4D, SLC44A1, SLC25A46, SNCAIP, SPTLC1, TBC1D2, TMEM175, TMEM38B, TMEM98, TRAK1, UBQLN1, UGT8, USP18, WDR36, XPA, XRCC1, ZSWIM6

Physiological System Development and Functions	p-value range	Molecules
Nervous System Development and Function	0.009-<0.0001	AGTPBP1, AP3B1, AP3S1, APC, ARHGEF28, C9orf72, CDK5R2, CDK7, CEP120, CPLANE1, DLG1, DNAJA1, DPYSL3, ELP1, EPG5, ERBIN, ERCC8, FER, FNTA, HMGCR, IL6ST, LPAR1, MAP3K1, NDUFS4, NIPBL, NTRK2, Pde4d, PDGFA, PELO, PIA2, PJA2, PLAA, PPARGC1A, PRKAA1, PTCH1, PTPRA, PTDRD, PTPRK, RASA1, RICTOR, RPS6, SEMA4D, SETBP1, SLC25A46, SNCAIP, THAP1, TMEM175, TMEM98, TRAK1, TRPM3, UGT8, XPA, XRCC1, ZSWIM6
Organ Morphology	0.009-<0.0001	ABCA1, AGTPBP1, AP3B1, APC, APOD, CDK5R2, cDK7, DPYSL3, ELAVL2, EPG5, ERCC8, GCK, GLIS3, HMGCR, MRAP, NDUFS4, NTRK2, PDGFA, PELO, PLIN2, PPARGC1A, PRKAA1, PTCH1, PTPRA, Ptprd, RASA1, RICTOR, RNLS, SLC16A2, THAP1, TMEM38B, UGT8, XPA, ZSWIM6
Organismal Development	0.009-<0.0001	ABCA1, AGTPBP1, APC, APOD, ARHGEF28, CDK5R2, CDK7, CENPN, CEP120, CPLANE1, DLG1, DNAJA1, DPYSL3, ELP1, EPG5, ERCC8, FER, FKTN, GCK, GLIS3, HMGCR, HSD17B4, IL6ST, LPAR1, MAP3K1, MRAP, NDUFS4, NIPBL, NTRK2, PDE4D, PDGFA, PELO, PIAS2, PIK3C3, PLAA, PLIN2, PPARGC1A, PRKAA1, PTCH1, PTPRA, Ptprd, PTPRK, RASA1, RIC1, RICTOR, RMI1, RNLS, RPS6, SEMA4D, SETBP1, SLC11A1, SLC16A2, SLC25A46, STARD4, TEAD1, THAP1, TLE1, TLE4, TMEM38B, TMEM98, TRAK1, UGT8, USP18, XPA, XRCC1, ZFAND5, ZSWIM6
Tissue Development	0.009-0.0001	ABCA1, AGTPBP1, APC, ARHGEF28, CATIP, CDK5R2, CERT1, CPLANE, DLG1, DPYSL3, FER, HMGCR, IL6ST, LPAR1, MAP3K1, MRAP, NIPBL, NPHS1, NTRK2, PDGFA, PIAS2, PJA2, PLAA, PPARGC1A, PRKAA1, PTPRA, Ptprd, PTPRK, RASA1, RICTOR, RPS6, SEMA4D, SLC16A2, SLC25A46, THAP1, TMEM38B, TMEM98, TRAK1, UGT8, XPA, XRCC1, ZFAND5, ZSWIM6
Tissue Morphology	0.009-0.0001	ABCA1, ACOT11, AGTPBP1, AP3B1, APC, CDK5R2, DPYSL3, ELP1, EPG5, ERBIN, ERCC8, FER, FKTN, HSD17B4, IL6ST, LPAR1, MAP3K1, MRAP, NDUFS4, NTRK2, PELO, PIK3C3, PLIN2, PPARGC1A, PRKAA1, PTCH1, PTPRA, Ptprd, RASA1, RICTOR, RPS6, SEMA4D, SNCAIP, SPTLC1, SSBP2, TEAD1, TMEM175, UBQLN1, UGT8, XPA, XRCC1, ZSWIM6

(c) Effect of sampling phase for male quail		
Molecular and Cellular Functions	p-value range	Molecules
Cellular Growth and Proliferation	0.0036-<0.0001	ACAN, ALK, ANLN, ANQ3, AQP1, CAMKK2, CHRNA1, CNP, DOCK1, DUSP10, EFHD1, ENPP2, EPAS1, FGR2, FKBP5, GAB1, GJC1, GPRC5B, HTRA1, MAP1B, MGLL, MITF, NR2E1, OBSCN, SEMA7A, SLC12A2, SLC1A2, SLC5A7, TENM3, TSPAN2, UGT8, ULK2, ZEB2
Cellular Development	0.0036-<0.0001	ACAN, ALK, ANQ3, AQP1, CAMKK2, CHRNA1, CNP, DOCK1, DUSP10, EFHD1, ENPP2, EPAS1, EPCAM, ERMIN, FGFR2, FKBP5, GAB1, GJC1, GPRC5B, XMOX1, MAP1B, MITF, MMD, NR2E1, OBSCN, SEMA7A, SLC12A2, SLC1A2, SLC5A7, TENM3, TSPAN2, UGT8, ULK2, ZEB2
Cellular Assembly and Organization	0.0035-<0.0001	ALK, APOH, AQP1, CAMKK2, CHRNA1, CNP, DIP2B, DOCK1, EFHD1, ENPP2, EPAS1, EPCAM, ERMIN, FGFR2, FKBP5, GAB1, GPRC5B, MAP1B, MGLL, NR2E1, RHOJ, SEMA7A, SGK2, SPATA13, TENM3, TSPAN2, UGT8, ULK2, ZEB2
Cellular Function and Maintenance	0.0038-<0.0001	ALK, AQP1, CAMKK2, CHRNA1, CLMP, CNP, DIP2B, DOCK1, DUSP10, EFHD1, ENPP2, EPAS1, EPCAM, ERMIN, FGFR2, GAB1, GPRC5B, HMOX1, LRRC8D, MAP1B, MGLL, MITF, NR2E1, NT5E, RHOJ, SEMA7A, SGK2, SLC12A2, SLC1A2, SLC4A4, SPATA13, TENM3, TP53BP2, TSPAN2, UGT8, ULK2, WIP1, ZEB2
Cell Morphology	0.0038-<0.0001	ALK, AQP1, CAMKK2, CHRNA1, CNP, DIP2B, DOCK1, EFHD1, ENPP2, EPCAM, ERMIN, FA2H, FGFR2, GAB1, GPRC5B, HMOX1, HTRA1, MAP1B, MGLL, MITF, NR2E1, NSD3, SEMA7A, SLC12A2, SPATA13, TENM3, TSPAN2, UGT8, ULK2, WIPF3, ZEB2

Physiological System Development and Functions	p-value range	Molecules
Tissue Development	0.0036-<0.0001	ACAN, ALK, AQP1, CAMKK2, CHRNA1, CNP, DOCK1, DUSP10, EFHD1, ENPP2, EPAS1, FGFRR2, FKBP5, GAB1, GJC1, GPRC5B, HMOX1, HTRA1, MAP1B, MITF, MMD, NR2E1, OBSCN, SEMA7A, SLC12A2, SLC1A2, SLC5A7, TENM3, TSPAN2, UGT8, ULK2, ZEB2
Nervous System Development and Function	0.0035-<0.0001	ACAN, ALK, AQP1, CAMKK2, CHRNA1, CNP, DIP2B, DOCK1, DUSP10, EFHD1, ENPP2, EPAS1, FA2H, FGFRR2, FKBP5, GAB1, GJC1, GPRC5B, HYCC1, MAP1B, MGLL, MITF, NR2E1, SEMA7A, SEPTIN5, SLC12A2, SLC5A7, TENM3, TP53BP2, TSPAN2, UGT8, ULK2, ZEB2
Organismal Development	0.0035-<0.0001	ALK, AQP1, CAMKK2, CHRNA1, CLMP, CNP, DOCK1, EFHD1, EPAS1, FA2H, FGFRR2, GAB1, GPRC5B, XMOX1, HTRA1, LDB3, MAP1B, MITF, NR2E1, OBSCN, RHOJ, SEMA7A, SLC12A2, SLC1A2, SLC4A4, SLC6A6, TENM3, TSPAN2, UGT8, ULK2, ZEB2
Digestive System Development and Function	0.0035-<0.0001	CLMP, FGFR2, SLC12A2, SLC4A4
Organ Morphology	0.0035-<0.0001	ALK, AQP1, CHRNA1, CLMP, FA2H, FGFRR2, XMOX1, LDB3, MAP1B, MITF, NR2E1, SEMA7A, SLC12A2, SLC1A2, SLC4A4, UGT8, WIPF3, ZEB2

The differences in gene expression patterns due to the overall effect of sex were predominantly associated with cellular assembly and organisation, as well as nervous system development (Table 2b). There was a total of 12 significant networks with scores ranging from 69 to 3 and numbers of focus genes ranging from 32 to 3 (the top 5 networks are shown in Table 2b). The top significant network mostly incorporated up-regulated genes in the males compared to the females and was associated with molecular transport and cellular organisation (Fig. S2, Supplementary Material 2).

**Table 3:** Top five IPA Networks using the gene lists of differentially expressed genes associated with (a) the overall effect of sampling phase, (b) sex, and (c) the effect of sampling phase for male quail.

**(a) Sampling phase**			
**Network**	**Score**	**Focus genes**	**Focus Genes**
1	28	10	APOH, CREM, ETNPPL, HOMER1, KAZALD1, LAMP2, RAB7B, SLC16A2, WTN4, ZNF398
2	7	3	PCDHGA5, RCHY1, TBC1D5

**(b) Sex**			
**Network**	**Score**	**Focus genes**	**Focus Genes**
1	69	32	ACAA2, AP3B1, ARL10, C9orf72, CLTA, DNAJA1, DPYSL3, GFM2, HOMER1, IPO11, LMBR1L, MRPS30, MTX3, NAA35, NDUFS4, NIPBL, NUP155, PELO, PIAS2, PJA2, PRAM1, PRKAA1, SLC25A46, SMARCA2, SPTLC1, TEAD1, TMEM41B, TNPO1, TRAK1, TRIM23, TXNL1, UBQLN1
2	46	24	ABCA1, ARSK, C18orf25, CH19, CENPN, ERBIN, FKTN, FUT10, METTL9, N-cor, Oasl2, PDZD2, PLIN2, PTPRA, Ptprd, PTPRK, SETBP1, SLC44A1, SSBP2, TBC1D2, TMEM38B, UBAP1, UGT8, ZBTB7C
3	46	24	ANO3, CCNH, CDK7, CEP120, DCP2, ELP1, ERCC8, EXOSC4, HSD17B4, HSDL2, HYLS1, NMRAL1, PCGF3, PLAA, POLR1B, RMI1, RNF20, SPIN1, TOPORS, UTP15, WDR36, XPA, XRCC1, ZFAND5
4	34	19	ANKRA2, CABP2, CDKR2, CERT1, FER, GLIS3, HMGCR, NTRK2, Pde4d, PDE4D, RGP1, RIC1, SMUI1, STARD4, THAP1, TLE1, TLE4, UBAP2, ZNF462
5	27	16	AP3S1, ATG10, C2CD2, CPLANE1, DCTN3, ELAV2, GPR22, LIPT2, MAST4, MED18, PHAX, PPWD1, TENT2, TRAPPC13, TTC33, ZNF608

**(c) Sampling phase for male quail**			
**Network**	**Score**	**Focus genes**	**Focus Genes**
1	50	22	ACAN, CAMKK2, CNP, ENPP2, EPAS1, EPCAM, ERMIN, FKBP5, HTRA1, MGLL, MMD, NTRA1, NR2E1, NT5E, SEMA7A, SGK2, SLC12A2, SLC1A2, SLC4A4, SLC5A7, UGT8, UVSSA,
2	44	20	ANLN, ANO3, APOH, AQP1, DIP2B, FAM102A, GAB1, INF2, INKA2, LRRC8D, MAP1B, NSD3, RTKN2, SEPTIN5, SEPTIN8, TP53BP2, TRIM25, USP54, WIPF3, WIPI1
3	31	15	ALK, CHRNA1, DOCK1, DUSP10, EFHD1, FGFR2, HMOX1, LDB3, MITF, PARP12, RHOJ, TENM3, TMEM140, TSPAN2, ZEB2
4	18	10	C10orf90, DCBLD1, ELOVL1, HAPLN2, KAZALD1, MOB3B, RILPL1, SLC6A6, TSPAN2, ULK2
5	18	10	C3orf20, CLDn20, CNOT9, ENPP6, FA2H, GPRC5B, HYCC1, PACS2, SPATA13, TXNDC16

There was a total of six distinct networks associated with the genes differentially expressed between migratory and non-migratory male samples, with scores ranging from 50 to 10 and with a number of focus genes from 22 to 6 (the focus genes in the top 5 networks are shown in Table 3c). The two most enriched networks incorporated a similarly large number of genes and were, therefore, merged together in order to achieve a broader and comprehensive view of the pathways involved (Fig. 3b). Most of the up-regulated genes in the males in a migratory state in relation to males in a non-migratory state were associated with cellular growth, tissue development and lipid metabolism (Table 2c).

### ddPCR gene expression analyses on selected genes

As expected, the two selected genes for ddPCR (APOH and LAMP2) showed concordant expression values and fold changes between the migratory and non-migratory state with the RNA-Sequencing data (Fig. S3, Supplementary Material 2), thus validating the transcriptomics results.

**Fig. 4:**
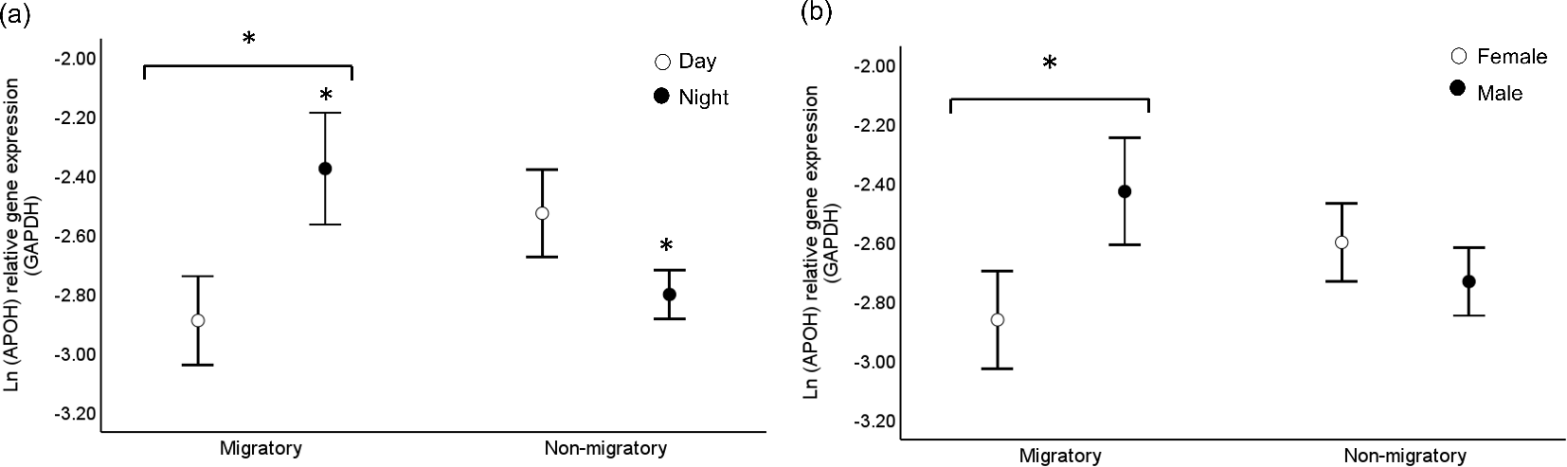
Hypothalamic APOH expression levels (data shown on the ln-scale) of Common quail differed in relation to (**a**) sampling phase (migratory or non-migratory) and sampling time (day or night), and in relation to (**b**) sampling phase and sex. (**a**) APOH expression levels were higher in the migratory birds sampled at night compared to the non-migratory birds sampled at night (*p* = 0.04); APOH expression levels did not differ between birds with different seasonal state sampled during the day (*p* = 0.06); within the migratory phase, APOH expression was higher in the birds sampled at night compared to the birds sampled during the day (*p* = 0.01), while no differences were detected between day and night sampling within the non-migratory birds (*p *= 0.16). (**b**) APOH expression levels were higher in the migratory males than the migratory females (*p* = 0.03) and no differences were found between non-migratory males and non-migratory females (*p* = 0.4); no differences on APOH expression levels were found between migratory and non-migratory birds within the same sex (*p* ≥ 0.12). * indicates *p* < 0.05. See Table S4a for full statistics. Data are shown as mean ± sem.

More importantly, when examining the expression levels of APOH across the entire study population, we consistently found this gene to be up-regulated in the migratory birds though such effect was dependent on the time of the day (sampling phase x sampling time interaction, *p* = 0.006 – full model output in Table S4a, Supplementary Material 2). In fact, the up-regulation of mRNA APOH was detected in the birds sampled at night and not in the birds sampled during the day (Fig. 4a), and this pattern was consistent for both sexes (Table S4a). The analyses further suggested that expression levels of APOH differed between male and female quail depending on the sampling phase (interaction: *p* = 0.04, Table S4a). This interaction was driven by higher APOH expression levels in the migratory males than the migratory females (Fig. 4b), while no differences were detected between the two sexes during the non-migratory sampling phase (Fig. 4b).

When examining the expression levels of LAMP2 in the entire study population, we found an interaction trend between sampling phase and sampling time (*p* = 0.07, full model output in Table S4b, Supplementary Material 2). Such effect was likely explained by the specific up-regulation of this gene in the migratory birds sampled at night compared to the non-migratory birds sampled at night (Fig. S4, Supplementary Material 2), corroborating the RNA-Seq results (Fig. 2). We found no differences in LAMP2 expression levels associated with sex either as main factor, or in interaction with the sampling phase or sampling time (Table S4b, Supplementary Material 2).

**Fig. 5:**
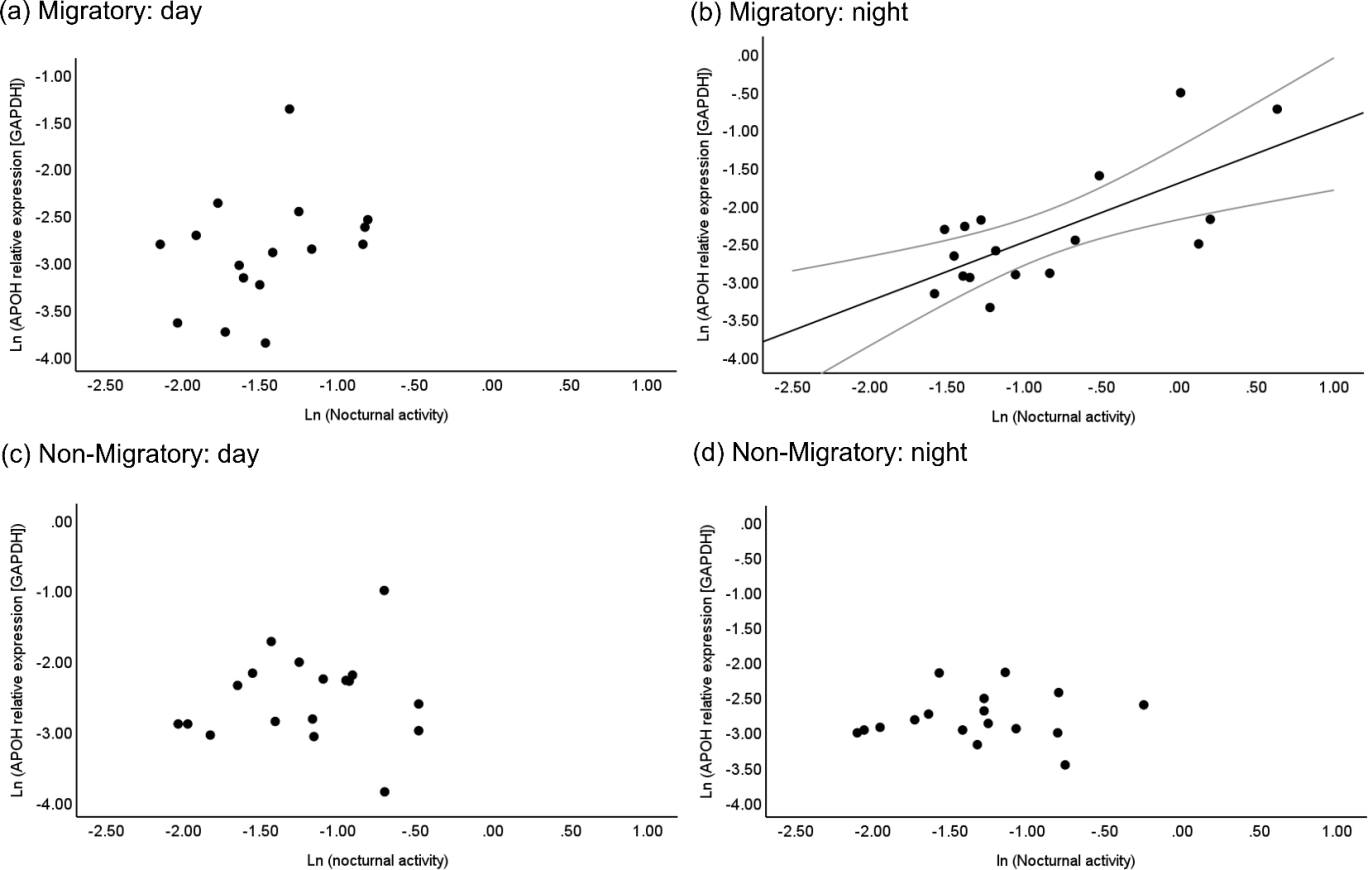
Hypothalamic APOH expression levels (data shown on the ln-scale) were positively associated with nocturnal activity (i.e. movements/min, data shown on the ln-scale) only in the migratory birds sampled (**b**) at night when restlessness (*p* = 0.01), and not in the migratory birds sampled during the day (**a**); no correlation was found in the non-migratory birds sampled either during the day (**c**), or the night (**d**). Grey lines in (**b**) represents 95% confidence interval. See Table S5 for full statistics.

Within the group of the birds sampled during the migratory phase and specifically at night, and not during the day, we additionally found a positive link between hypothalamic APOH expression levels and nocturnal restlessness (*p* = 0.01, full model output in Table S5a, Supplementary Material 2; Fig. 5a-b); the correlation between activity levels and APOH was absent within the birds sampled during the non-migratory phase, either during the day or during the night (Table S5a; Fig. 5c-d). We found no link between mRNA APOH and levels of food intake within both sampling phases. Neither levels of food intake nor nocturnal restlessness were linked with hypothalamic LAMP2 expression levels in either the migratory, or non-migratory birds (Table S5b and Figure S5a-d, Supplementary Material 2).

## Discussion

In this study we controlled the seasonal transition of Common quails by simulating an autumn migratory state followed by a non-migratory state. We first examined global gene expression changes within the brain (hypothalamus) of quails sampled at a standardised night-time either in the migratory state, or non-migratory state using RNA-Sequencing. All birds experienced the same photoperiod conditions at the time of sample collection (12:12 hrs light:dark cycle). This enabled us to rule out the possibility that gene expression differences between the two seasonal states could be due to direct differences in day length exposure, which can trigger rapid and marked changes in the brain transcriptome as shown in the migratory Black-headed bunting^26^. These RNA-Seq analyses were selectively performed in those birds showing the most divergent seasonal phenotypes in relation to levels of nocturnal restlessness and fattening rates, which are well recognised key proxies of the migratory state. The main results from these genome-wide analyses were that: (i) regardless of the sex, specific functional pathways targeting lipidic trafficking, molecular transport, and endocrine functioning were associated with the migratory state, and (ii) these seasonal transcriptome-dependent changes appeared at higher magnitude and more enriched in males compared to females. Our follow-up experiment on two functionally relevant target genes (apolipoprotein H or APOH and lysosomal associated membrane protein 2 or LAMP2) validated the RNA-Sequencing results and additionally suggested that their up-regulation during the migratory state occurred specifically at night and not during the day. Importantly, we also found that the up-regulation of APOH was positively linked with levels of nocturnal restlessness within the migratory sampling phase at night; while such a correlation was absent within the non-migratory sampling phase regardless of time of sampling. Results focusing on the few candidate pathways are discussed in more detail below.

We found relatively few genes differentially expressed in relation to the migratory state (*n*= 16), a number even lower than has been reported in previous work in migratory birds using high-throughput gene expression sequencing in brain-derived tissues^24,57^. However, these differentially expressed genes were clearly associated with enriched up-regulation of functional pathways targeting fatty acid and carbohydrate metabolism, molecular transport, behavioural modulation, and endocrine system synthesis as highlighted by the core functional analyses. Our data are broadly consistent with previous work in migratory birds that also found changes in gene transcripts regulating metabolic pathways for lipids, carbohydrates and proteins within the brain as well as peripheral organs such as the liver and the heart^24,57–60^. These results are not surprising and most likely attributable to hyperphagia and the enhanced metabolic demands that is typical of birds in a migratory state^5,61^.

A result of particular interest was the up-regulation of APOH and LAMP2 and their clustering together in the top significant functional network (Fig. 3a). Apolipoproteins, including the class APOH, are synthesised in various organs and have a well-established major role in the transport of lipids and cholesterol within the central nervous system and the periphery^62–64^. In humans, APOH is synthesised within the brain but can also enter the brain as “lipid-poor” particle or as free low-density apolipoprotein^65^. Concentrations levels of APOH in the plasma are positively associated with triglycerides and triglyceride-rich lipoproteins, and are considered a clinical marker of insulin resistance^66^. LAMP2 is a gene belonging to the family of membrane glycoproteins. LAMP2 can directly bind to fatty molecules and is involved in lysosomal cholesterol dissemination^67^, thus promoting the process of lipophagy^68^.

Apolipoproteins (apolipoprotein AI and apolipoprotein O) were also found highly expressed in the brain transcriptome of migratory wheatears^24^, and within the liver, transcripts involved in lipid transport (including apolipoprotein A1) were generally increasing as birds were accumulating fat stores^59^. Altogether, these data suggest that apolipoprotein pathways may be key processes integrating signals between the central nervous system and the periphery allowing migratory birds to limit fat tissue overloading and activate the transport and utilization of fatty acids. This possibility is further supported by two complementary results: (1) that we detected an up-regulation of hypothalamic APOH specifically at night and only during the migratory phase, and, importantly, (2) that within this phase, mRNA hypothalamic APOH was associated with levels of nocturnal restlessness with the birds showing higher expression levels of APOH being those birds most active at night.

Fatty acids are the main energy source during endurance migratory flights^5^. Due to their highly hydrophobic nature, however, they have to be actively transported from the adipose tissue and liver through the circulation into the muscles where they are oxidised. Thus, migratory birds are under strong selective pressures to produce fatty acid carriers^69,70^. This contrasts with mammals that cannot burn fat at high intensity exercise presumably due to low investments in fat trafficking mechanisms^61^. Circulatory transport of fatty acids appears accelerated during the nocturnal migratory flight in some passerine species via a lipoprotein-mediated pathway that would primarily involve low density classes of lipoproteins^69^. Our data, linking hypothalamic expression of APOH with nocturnal restlessness, provide novel experimental findings supporting such a hypothesis. Tissue-specific and carefully designed omics experiments would be necessary in the future to elucidate how pathways regulating fatty acid transport and utilization are co-ordinated between the nervous central system and peripheral organs and how such pathways contribute to stimulation of energy turnovers triggering migratory movements.

Our analyses showed that 97 genes were differentially expressed due to potential interactive effects between the migratory state and the sex. These results suggest that certain genes are differentially expressed while males are in a migratory state, and that these are distinct from those genes expressed in the females either in a migratory or non-migratory state. We specifically focused on disentangling possible differences in gene expression between migratory and non-migratory birds within the same sex. This analysis highlighted a larger number of genes differentially expressed when comparing migratory and non-migratory males (*n* = 93), than between migratory and non-migratory females (*n* = 4). However, the top two differentially expressed gene networks within the male samples (Fig. 3b) did not appear much different from the top network associated with the migratory state as main factor (Fig. 3a). In fact, the gene networks shown in Fig. 3b once again included APOH and incorporated the ERK (Extracellular signal-regulated kinase), a canonical pathway implicated in tissue regeneration in vertebrates^71^, and the NFKb, an important complex regulator of synaptic plasticity and memory^72,73^. The network of functionally associated genes was more strongly enriched with genes implicated in fatty acid metabolism, which additionally included FAR2 (fatty acyl-CoA reductase 2), FA2H (fatty acid 2-hydroxylase), and ELOV1 (fatty acid elongase 1). It also included the EPAS1 gene that is associated with power athletic performance in humans^74^and was found consistently differentially expressed in migratory birds sampled under differing fattening states in the brain and liver^24,59^, and in the whole blood^75^. The network analyses also suggested that the mapped focus genes were indirectly linked to glucocorticoid regulation, growth hormone and insulin complex, which are physiologically conserved regulators of body growth and energy homeostasis. In hibernating mammals, naturally induced reversible insulin resistance is a key mediator for the development of pre-hibernation fattening (reviewed by^76^). Glucose homeostasis in birds differs greatly from mammals as they show adaptations towards insulin resistance to keep continuously high glucose circulating levels possibly to meet the high metabolic demands of flight^77^. However, glucose regulation appears to change seasonally also in birds^78,79^and whether changes in insulin sensitivity might drive pre-migratory fattening remains completely untested. The suggested involvement of the growth hormone is somewhat linked with our previous findings in the same study population showing that the birds in migratory state had elevated concentrations of plasma ghrelin compared to birds in a non-migratory state^33^. However we have no information on how exactly the ghrelin axis is centrally regulated in birds to be able to draw conclusive statements on possible mechanisms. Research testing if and how growth hormone/insulin-related signalling pathways influence migratory life history strategies remains surprisingly little explored, but clearly crucially needed^80,81^.

The sex-specific differences in the migratory state we found might be explained by differences in the timing to migrate and/or fuelling dynamics between the two sexes of Common quail. In fact, our photoperiod protocol, to simulate seasonal transitioning, did lead to steeper decreases in body mass and fat stores in the males compared to the females^32^. Protandry - the earlier arrival of males in the breeding sites – is a well-known phenomenon in the Common quail^82,83^but little is known about sex-specific differences in the onset of migration during autumn^84^. Ringing data of wild quails captured along the northern cost of Sinai during the autumn passage showed differences in sex ratio over the course of migratory waves and suggested earlier captures of migrating males compared to females^85^. Overall, our data highlight the importance of considering sex-specific factors as possible modulators of seasonal migrations. This is an aspect which is often neglected in avian research. Future studies, ideally in wild populations captured along the migratory flyway passages and employing whole blood transcriptomics^75,86^, may prove very useful in this context.

We also found large differences in the brain transcriptome of quails due to sex as a main factor (*n*= 225 differentially expressed genes). These differences were skewed towards gene expression up-regulation in the males compared to females and were broadly associated with cellular organisation and nervous system functioning. Sexual dimorphism in aromatase activity has been well characterised in the brain of the Japanese quail and is thought to play an important role in the activation by testosterone of male sexual behaviour^87^. Our functional analyses does not point to specific differentially expressed endocrine/steroid-related pathways between the two sexes. This is not surprising as all the samples were taken from quails that never experienced gonadal development before and throughout the entire experiment. The main focus of this study was not to examine changes in gene expression pathways between male and female *per se*, but rather to examine expression changes in relation to the migratory state while controlling for the presence of the two sexes.

To conclude, findings from this study provide new insights into seasonal-related changes in the brain transcriptome that were directly linked with proxies of the migratory state. Our data are broadly consistent with previous findings in the brain and peripheral organs of the studied songbirds as we found up-regulation of genes involved in lipid trafficking and fatty acid utilisation. More importantly, our study provides novel experimental findings revealing that neuronal functional pathways mediating the circulatory transport of lipids likely represent key adaptive physiological processes to activate fat breakdown and thus promote nocturnal migratory movements in birds. This neglected hypothesis developed from intriguing findings from earlier work^69^ now, critically, needs deeper future investigations to unveil the exact functional mechanisms.

## Electronic supplementary material

Below is the link to the electronic supplementary material.

Supplementary Material 1

Supplementary Material 2

## Data Availability

Raw RNA-Sequencing data and assembly file are available from the Sequence Read Archive (SRA) on NCBI, study accession number PRJNA630138 (https://www.ncbi.nlm.nih.gov/bioproject/PRJNA630138/). The original contributions presented in the study are included in the Supplementary Materials.

## References

[1] Bairlein, F. How to get fat: nutritional mechanisms of seasonal fat accumulation in migratory songbirds. *Naturwissenschaften*. **89**, 1–10. 10.1007/s00114-001-0279-6 (2002). 12008967 10.1007/s00114-001-0279-6

[2] Berthold, P. Evolutionary aspects of migratory behavior in European warblers. *J. Evol. Biol.* **1**, 195–209. 10.1046/j.1420-9101.1998.1030195.x (1988).

[3] Piersma, T., Perez-Tris, J., Mouritsen, H., Bauchinger, U. & Bairlein, F. Is there a migratory syndrome common to all migrant birds? *Ann. N Y Acad. Sci.* **1046**, 282–293. 10.1196/annals.1343.026 (2005). 16055861 10.1196/annals.1343.026

[4] Newton, I. *Bird Migration*HarperCollins,. (2010).

[5] Guglielmo, C. G. Obese super athletes: fat-fueled migration in birds and bats. *J. Exp. Biol.* **221**, jeb165753. 10.1242/jeb.165753 (2018). 29514885 10.1242/jeb.165753

[6] Rattenborg, N. C. et al. Migratory sleeplessness in the White-Crowned Sparrow (*Zonotrichia leucophrys gambelii*). *PLoS Biol.* **2**, e212. 10.1371/journal.pbio.0020212 (2004). 15252455 10.1371/journal.pbio.0020212 PMC449897

[7] Berthold, P. *Control of bird Migration* (Chapman & Hall, 1996).

[8] Gwinner, E. in *In Adv. Stud. Behav*. Vol. 16, 191–228 (eds Rosenblatt, J. S., Beer, C., Busnel, M. C. & Slater, P. J. B.) (Academic, 1986).

[9] Lupi, S., Slezacek, J. & Fusani, L. The physiology of stopover decisions: food, fat and zugunruhe on a Mediterranean island. *J. Ornithol.* **160**, 1205–1212. 10.1007/s10336-019-01693-4 (2019).

[10] Gwinner, E. in *In Bird Migration. Physiology and Ecophysiology*. 257–268 (eds Gwinner, E.) (Springer, 1990).

[11] Helm, B. & Gwinner, E. Migratory restlessness in an equatorial nonmigratory bird. *PLoS Biol.* **4**, e110. 10.1371/journal.pbio.0040110 (2006). 16555925 10.1371/journal.pbio.0040110 PMC1420642

[12] Williams, B. K., Nichols, J. D. & Conroy, M. J. *Analysis and Management of Animal Populations. Modeling, Estimation, and Decision Making* (Academic, 2002).

[13] Helbig, A. J. Inheritance of migratory direction in a bird species: a cross-breeding experiment with SE- and SW-migrating blackcaps (Sylvia atricapilla). *Behav. Ecol. Sociobiol.* **28**, 9–12 (1991).

[14] Derégnaucourt, S., Guyomarc’h, J. C. & Spanò, S. Behavioural evidence of hybridization (Japanese×European) in domestic quail released as game birds. *Appl. Anim. Behav. Sci.* **94**, 303–318. 10.1016/j.applanim.2005.03.002 (2005).

[15] Liedvogel, M., Åkesson, S. & Bensch, S. The genetics of migration on the move. *Trends Ecol. Evol.* **26**, 561–569. 10.1016/j.tree.2011.07.009 (2011). 21862171 10.1016/j.tree.2011.07.009

[16] Mueller, J. C., Pulido, F. & Kempenaers, B. Identification of a gene associated with avian migratory behaviour. *Proceedings of the Royal Society B-Biological Sciences* 278, 2848–2856, doi: (2011). 10.1098/rspb.2010.2567 10.1098/rspb.2010.2567 PMC3145181 21325325

[17] Saino, N. et al. Polymorphism at the *clock* gene predicts phenology of long-distance migration in birds. *Mol. Ecol.* **24**, 1758–1773. 10.1111/mec.13159 (2015). 25780812 10.1111/mec.13159

[18] Sanchez-Donoso, I. et al. Massive genome inversion drives coexistence of divergent morphs in common quails. *Curr. Biol.* **32**, 462–469e466. 10.1016/j.cub.2021.11.019 (2022). 34847353 10.1016/j.cub.2021.11.019

[19] Sokolovskis, K. et al. Migration direction in a songbird explained by two loci. *Nat. Commun.* **14**, 165. 10.1038/s41467-023-35788-7 (2023). 36631459 10.1038/s41467-023-35788-7 PMC9834303

[20] Gu, Z. et al. Climate-driven flyway changes and memory-based long-distance migration. *Nature*. **591**, 259–264. 10.1038/s41586-021-03265-0 (2021). 33658718 10.1038/s41586-021-03265-0

[21] Pulido, F., Berthold, P. & van Noordwijk, A. J. Frequency of migrants and migratory activity are genetically correlated in a bird population: Evolutionary implications. *Proc. Natl. Acad. Sci. USA* 93, 14642–14647 (1996). 10.1073/pnas.93.25.14642 PMC26188 8962107

[22] Cornelius, J. M., Boswell, T., Jenni-Eiermann, S., Breuner, C. W. & Ramenofsky, M. Contributions of endocrinology to the migration life history of birds. *Gen. Comp. Endocrinol.* **190**, 47–60. 10.1016/j.ygcen.2013.03.027 (2013). 23602795 10.1016/j.ygcen.2013.03.027

[23] Boswell, T. & Dunn, I. C. Regulation of Agouti-related protein and Pro-opiomelanocortin Gene expression in the Avian Arcuate Nucleus. *Front. Endocrinol.* **8**10.3389/fendo.2017.00075 (2017). 10.3389/fendo.2017.00075 PMC5389969 28450851

[24] Frias-Soler, R. C., Pildaín, L. V., Pârâu, L. G., Wink, M. & Bairlein, F. Transcriptome signatures in the brain of a migratory songbird. *Comp. Biochem. Physiol. D: Genomics Proteomics*. **34**, 100681. 10.1016/j.cbd.2020.100681 (2020). 32222683 10.1016/j.cbd.2020.100681

[25] Johnston, R. A., Paxton, K. L., Moore, F. R., Wayne, R. K. & Smith, T. B. Seasonal gene expression in a migratory songbird. *Mol. Ecol.* **25**, 5680–5691. 10.1111/mec.13879 (2016). 27747949 10.1111/mec.13879

[26] Sharma, A. et al. Photoperiodically driven transcriptome-wide changes in the hypothalamus reveal transcriptional differences between physiologically contrasting seasonal life-history states in migratory songbirds. *Sci. Rep.* **11**, 12823. 10.1038/s41598-021-91951-4 (2021). 34140553 10.1038/s41598-021-91951-4 PMC8211672

[27] Boss, J. et al. Gene expression in the brain of a migratory songbird during breeding and migration. *Mov. Ecol.* **4**. 10.1186/s40462-016-0069-6 (2016). 10.1186/s40462-016-0069-6 PMC4753645 26881054

[28] Marasco, V., Herzyk, P., Robinson, J. & Spencer, K. A. Pre- and post-natal stress programming: developmental exposure to glucocorticoids causes long-term brain-region specific changes to Transcriptome in the precocial Japanese quail. *J. Neuroendocrinol.* **28**. 10.1111/jne.12387 (2016). 10.1111/jne.12387 PMC5103168 26999292

[29] Naurin, S., Hansson, B., Hasselquist, D., Kim, Y. H. & Bensch, S. The sex-biased brain: sexual dimorphism in gene expression in two species of songbirds. *BMC Genom.* **12**, 37. 10.1186/1471-2164-12-37 (2011). 10.1186/1471-2164-12-37 PMC3036617 21235773

[30] Patchett, R., Kirschel, A. N. G., King, R., Styles, J., Cresswell, W. & P. & Age-related changes in migratory behaviour within the first annual cycle of a passerine bird. *PLoS ONE*. **17**, e0273686. 10.1371/journal.pone.0273686 (2022). 36260548 10.1371/journal.pone.0273686 PMC9581414

[31] Boswell, T., Hall, M. R. & Goldsmith, A. R. Annual cycles of migratory fattening, reproduction and moult in European quail (*Coturnix coturnix*). *J. Zool.* **231**, 627–644. 10.1111/j.1469-7998.1993.tb01943.x (1993).

[32] Marasco, V., Sebastiano, M., Costantini, D., Pola, G. & Fusani, L. Controlled expression of the migratory phenotype affects oxidative status in birds. *J. Exp. Biol.* **224**, jeb233486. 10.1242/jeb.233486 (2021). 33536304 10.1242/jeb.233486

[33] Marasco, V., Kaiya, H., Pola, G. & Fusani, L. Ghrelin, not corticosterone, is associated with transitioning of phenotypic states in a migratory Galliform. *Front. Endocrinol.* **13**, 1058298. 10.3389/fendo.2022.1058298 (2023). 10.3389/fendo.2022.1058298 PMC9869107 36699038

[34] Robinson, J. E. & Follett, B. K. Photoperiodism in Japanese quail: the termination of Seasonal breeding by Photorefractoriness. *Proc. Royal Soc. Lond. Ser. B Biol. Sci.* **215**, 95–116 (1982). 10.1098/rspb.1982.0030 6127699

[35] Marasco, V., Sebastiano, M., Costantini, D., Pola, G. & Fusani, L. Controlled expression of the migratory phenotype affects oxidative status in birds. *The Journal of Experimental Biology*, jeb.233486, doi: (2021). 10.1242/jeb.233486 10.1242/jeb.233486 33536304

[36] Smith, S., Fusani, L., Boglarka, B., Sanchez-Donoso, I. & Marasco, V. Lack of introgression of Japanese quail in a captive population of common quail. *Eur. J. Wildl. Res.* **64**, 51. 10.1007/s10344-018-1209-7 (2018).

[37] Ouyang, J. Q., Davies, S. & Dominoni, D. Hormonally mediated effects of artificial light at night on behavior and fitness: linking endocrine mechanisms with function. *J. Exp. Biol.* **221**10.1242/jeb.156893 (2018). 10.1242/jeb.156893 PMC5897701 29545373

[38] Sachs, B. D. Photoperiodic control of reproductive behavior and physiology of the male Japanese quail (Coturnix coturnix japonica). *Horm. Behav.* **1**, 7–24. 10.1016/0018-506X(69)90002-6 (1969).

[39] DERÉGNAUCOURT, S., GUYOMARC’H, J. C. & BELHAMRA, M. Comparison of migratory tendency in European quail Coturnix c. coturnix, domestic Japanese quail Coturnix c. Japonica and their hybrids. *Ibis*. **147**, 25–36. 10.1111/j.1474-919x.2004.00313.x (2005).

[40] Marasco, V., Fusani, L., Pola, G. & Smith, S. Data on the de novo transcriptome assembly for the migratory bird, the common quail (Coturnix coturnix). *Data Brief.* **32**, 106041. 10.1016/j.dib.2020.106041 (2020). 32775561 10.1016/j.dib.2020.106041 PMC7394769

[41] Puelles, L., Martinez-de-la-Torre, M., Paxinos, G., Watson, C. & Martinez, S. *The Chick Brain in Stereotaxic Coordinates: An Atlas Featuring Neuromeric Subdivisions and Mammalian Homologies* (Academic, 2007).

[42] Lessells, C. M. & Boag, P. T. Unrepeatable repeatabilities - A Common Mistake. *Auk*. **104**, 116–121 (1987).

[43] Bertin, A., Houdelier, C., Richard-Yris, M. A., Guyomarc’h, C. & Lumineau, S. Stable individual profiles of daily timing of migratory restlessness in European quail. *Chronobiol Int.* **24**, 253–267. 10.1080/07420520701283685 (2007). 17453846 10.1080/07420520701283685

[44] Zúñiga, D. et al. Abrupt switch to migratory night flight in a wild migratory songbird. *Sci. Rep.* **6**, 34207. 10.1038/srep34207 (2016). 27666200 10.1038/srep34207 PMC5035921

[45] Chen, T. H., Gross, J. A. & Karasov, W. H. Chronic exposure to pentavalent arsenic of larval leopard frogs (Rana pipiens): bioaccumulation and reduced swimming performance. *Ecotoxicology*. **18**, 587–593. 10.1007/S10646-009-0316-3 (2009). 19396542 10.1007/s10646-009-0316-3

[46] Bryant, D. M. et al. A tissue-mapped Axolotl De Novo Transcriptome enables identification of limb regeneration factors. *Cell. Rep.* **18**, 762–776. 10.1016/j.celrep.2016.12.063 (2017). 28099853 10.1016/j.celrep.2016.12.063 PMC5419050

[47] Powell, S. et al. eggNOG v3.0: orthologous groups covering 1133 organisms at 41 different taxonomic ranges. *Nucleic Acids Res.* **40**, D284–D289. 10.1093/nar/gkr1060 (2012). 22096231 10.1093/nar/gkr1060 PMC3245133

[48] Conesa, A. et al. A survey of best practices for RNA-seq data analysis. *Genome Biol.* **17**10.1186/s13059-016-0881-8 (2016). 10.1186/s13059-016-0881-8 PMC4728800 26813401

[49] Bolger, A. M., Lohse, M. & Usadel, B. Trimmomatic: a flexible trimmer for Illumina sequence data. *Bioinformatics*. **30**, 2114–2120. 10.1093/bioinformatics/btu170 (2014). 24695404 10.1093/bioinformatics/btu170 PMC4103590

[50] Bray, N. L., Pimentel, H., Melsted, P. & Pachter, L. Near-optimal probabilistic RNA-seq quantification. *Nat. Biotechnol.* **34**, 525–527. 10.1038/nbt.3519 (2016). 27043002 10.1038/nbt.3519

[51] Love, M. I., Huber, W. & Anders, S. Moderated estimation of Fold change and dispersion for RNA-seq data with DESeq2. *Genome Biol.* **15**, 550. 10.1186/s13059-014-0550-8 (2014). 25516281 10.1186/s13059-014-0550-8 PMC4302049

[52] Benjamini, Y. & Hochberg, Y. Controlling the false Discovery rate: a practical and powerful Approach to multiple testing. *J. R Stat. Soc. B*. **57**, 289–300. 10.2307/2346101 (1995).

[53] R: A language and environment for statistical computing. R Foundation for Statistical Computing, Vienna, Austria, (2022).

[54] RStudio & RStudio Integrated Development for R (PBC, Boston, MA, (2023).

[55] emmeans: Estimated Marginal Means, aka Least-Squares Means. R package version 1.8.9. (2023).

[56] Zuur, A. F., Ieno, E. N., Walker, N. J., Saveliev, A. A. & Smith, G. M. *Mixed Efffects Models and Extensions in Ecology with R*. I-XXII, 1-574; Index (Springer, 2009).

[57] Sharma, A., Singh, D., Das, S. & Kumar, V. Hypothalamic and liver transcriptome from two crucial life-history stages in a migratory songbird. *Exp. Physiol.* **103**, 559–569. 10.1113/EP086831 (2018). 29380464 10.1113/EP086831

[58] Singh, D., Swarup, V., Le, H. & Kumar, V. Transcriptional Signatures in liver reveal metabolic adaptations to Seasons in Migratory Blackheaded Buntings. *Front. Physiol.* **9**, 1568. 10.3389/fphys.2018.01568 (2018). 30538637 10.3389/fphys.2018.01568 PMC6277527

[59] Frias-Soler, R. C., Kelsey, N. A., Villarín Pildaín, L., Wink, M. & Bairlein, F. Transcriptome signature changes in the liver of a migratory passerine. *Genomics*. **114**, 110283. 10.1016/j.ygeno.2022.110283 (2022). 35143886 10.1016/j.ygeno.2022.110283

[60] Horton, W. J. et al. Transcriptome analyses of Heart and Liver Reveal Novel pathways for regulating Songbird Migration. *Sci. Rep.* **9**, 6058. 10.1038/s41598-019-41252-8 (2019). 30988315 10.1038/s41598-019-41252-8 PMC6465361

[61] Guglielmo, C. G. Move that fatty acid: fuel selection and transport in migratory birds and bats. *Integr. Comp. Biol.* **50**, 336–345. 10.1093/icb/icq097 (2010). 21558208 10.1093/icb/icq097

[62] Elliott, D. A., Weickert, C. S. & Garner, B. Apolipoproteins in the brain: implications for neurological and psychiatric disorders. *Clin. Lipidol.* **5**, 555–573. 10.2217/clp.10.37 (2010). 10.2217/CLP.10.37 PMC3058497 21423873

[63] Mather, K. A. et al. Genome-wide significant results identified for plasma apolipoprotein H levels in middle-aged and older adults. *Sci. Rep.* **6**, 23675. 10.1038/srep23675 (2016). 27030319 10.1038/srep23675 PMC4814826

[64] Mahley, R. W. & Apolipoprotein, E. Cholesterol Transport Protein with Expanding Role in Cell Biology. *Science*. **240**, 622–630. 10.1126/science.3283935 (1988). 3283935 10.1126/science.3283935

[65] Koch, S. et al. Characterization of four lipoprotein classes in human cerebrospinal fluid. *J. Lipid Res.* **42**, 1143–1151. 10.1016/S0022-2275(20)31605-9 (2001). 11441143

[66] Castro, A. et al. APOH is increased in the plasma and liver of type 2 diabetic patients with metabolic syndrome. *Atherosclerosis*. **209**, 201–205. 10.1016/j.atherosclerosis.2009.09.072 (2010). 19878946 10.1016/j.atherosclerosis.2009.09.072

[67] Li, J. & Pfeffer, S. R. Lysosomal membrane glycoproteins bind cholesterol and contribute to lysosomal cholesterol export. *eLife*. **5**, e21635. 10.7554/eLife.21635 (2016). 27664420 10.7554/eLife.21635 PMC5068966

[68] Gu, J. et al. The role of lysosomal membrane proteins in glucose and lipid metabolism. *FASEB J.* **35**, e21848. 10.1096/fj.202002602R (2021). 34582051 10.1096/fj.202002602R

[69] Jenni-Eiermann, S. & Jenni, L. High plasma triglyceride levels in small birds during migratory flight: a new pathway for fuel supply during endurance locomotion at very high Mass-Specific Metabolic Rates? *Physiol. Zool.* **65**, 112–123. 10.1086/physzool.65.1.30158242 (1992).

[70] Vock, R. et al. Design of the oxygen and substrate pathways: V. Structural basis of vascular substrate supply to muscle cells. *J. Exp. Biol.* **199**, 1675–1688. 10.1242/jeb.199.8.1675 (1996). 8708575 10.1242/jeb.199.8.1675

[71] Wen, X., Jiao, L. & Tan, H. MAPK/ERK Pathway as a Central Regulator in Vertebrate Organ Regeneration. *Int. J. Mol. Sci.* **23**, 1464 (2022). 35163418 10.3390/ijms23031464 PMC8835994

[72] Meffert, M. K., Chang, J. M., Wiltgen, B. J., Fanselow, M. S. & Baltimore D. NF-κB functions in synaptic signaling and behavior. *Nat. Neurosci.* **6**, 1072–1078. 10.1038/nn1110 (2003). 12947408 10.1038/nn1110

[73] Mattson, M. P. & Meffert, M. K. Roles for NF-κB in nerve cell survival, plasticity, and disease. *Cell. Death Differ.* **13**, 852–860. 10.1038/sj.cdd.4401837 (2006). 16397579 10.1038/sj.cdd.4401837

[74] Voisin, S. et al. EPAS1 gene variants are associated with sprint/power athletic performance in two cohorts of European athletes. *BMC Genom.* **15**, 382. 10.1186/1471-2164-15-382 (2014). 10.1186/1471-2164-15-382 PMC4035083 24884370

[75] Bounas, A. et al. Adaptive regulation of stopover refueling during Bird Migration: insights from whole blood transcriptomics. *Gen. Biol. Evol.* **15**, evad061. 10.1093/gbe/evad061 (2023). 10.1093/gbe/evad061 PMC10139441 37067540

[76] Wu, C. W., Biggar, K. K. & Storey, K. B. Biochemical adaptations of mammalian hibernation: exploring squirrels as a perspective model for naturally induced reversible insulin resistance. *Braz. J. Med. Biol. Res.* **46**, 1–13. 10.1590/1414-431x20122388 (2013). 23314346 10.1590/1414-431X20122388 PMC3854349

[77] Satoh, T. Bird evolution by insulin resistance. *Trends Endocrinol. Metab.* **32**, 803–813. 10.1016/j.tem.2021.07.007 (2021). 34446347 10.1016/j.tem.2021.07.007

[78] Gupta, N. J., Nanda, R. K., Das, S., Das, M. K. & Arya, R. Night migratory songbirds exhibit metabolic ability to Support High Aerobic Capacity during Migration. *ACS Omega*. **5**, 28088–28095. 10.1021/acsomega.0c03691 (2020). 33163791 10.1021/acsomega.0c03691 PMC7643192

[79] Sweazea, K. L., Tsosie, K. S., Beckman, E. J., Benham, P. M. & Witt, C. C. Seasonal and elevational variation in glucose and glycogen in two songbird species. *Comp. Biochem. Physiol. A*. **245**, 110703. 10.1016/j.cbpa.2020.110703 (2020). 10.1016/j.cbpa.2020.110703 32283178

[80] Price, E. R. et al. Migration- and exercise-induced changes to flight muscle size in migratory birds and association with IGF1 and myostatin mRNA expression. *J. Exp. Biol.* **214**, 2823–2831. 10.1242/jeb.057620 (2011). 21832125 10.1242/jeb.057620

[81] Pradhan, D. S., Ma, C., Schlinger, B. A., Soma, K. K. & Ramenofsky, M. Preparing to migrate: expression of androgen signaling molecules and insulin-like growth factor-1 in skeletal muscles of Gambel’s white-crowned sparrows. *J. Comp. Physiol. A*. **205**, 113–123. 10.1007/s00359-018-1308-7 (2019). 10.1007/s00359-018-1308-7 30535830

[82] Puigcerver, M. *Contribución al conocimiento de la biología y ecoetología de la codorniz (Coturnix coturnix)* phd thesis, University of Barcelona, (1991).

[83] Zduniak, P. & Yosef, R. Age and sex determine the phenology and biometrics of migratory common quail (*Coturnix coturnix*) at Eilat, Israel. *Ornis Fennica*. **85**, 37–45 (2008).

[84] Perennou, C. *European Union Management Plan 2009–2011. Common Quail Coturnix coturnix* (European Commission, 2009).

[85] Zuckerbrot, Y. D., Safriel, U. N. & Paz, U. Autumn migration of quail *Coturnix coturnix* at the north coast of the Sinai Peninsula. *Ibis*. **122**, 1–14. 10.1111/j.1474-919X.1980.tb00867.x (1980).

[86] Franchini, P. et al. Animal tracking meets migration genomics: transcriptomic analysis of a partially migratory bird species. *Mol. Ecol.* **26**, 3204–3216. 10.1111/mec.14108 (2017). 28316119 10.1111/mec.14108

[87] Balthazart, J., Tlemçani, O. & Ball, G. F. Do sex differences in the brain explain sex differences in the Hormonal induction of Reproductive Behavior? What 25 years of Research on the Japanese quail tells us. *Horm. Behav.* **30**, 627–661. 10.1006/hbeh.1996.0066 (1996). 9047287 10.1006/hbeh.1996.0066

