# Publisher Correction: Brain gene expression reveals pathways underlying nocturnal migratory restlessness

**DOI:** 10.1038/s41598-024-80825-0

**Published:** 2024-12-03

**Authors:** Valeria Marasco, Leonida Fusani, Patricia Haubensak, Gianni Pola, Steve Smith

**Affiliations:** 1 Department of Interdisciplinary Life Sciences, Research Institute of Wildlife Ecology, University of Veterinary Medicine Vienna, Savoyenstraße 1a, Vienna, 1160 Austria; 2 Department of Interdisciplinary Life Sciences, Konrad Lorenz Institute of Ethology, University of Veterinary Medicine Vienna, Savoyenstraße 1a, Vienna, A-1160 Austria; 3 Department of Behavioural and Cognitive Biology, University of Vienna, Djerassiplatz 1, Vienna, 1030 Austria; 4 Istituto Sperimentale Zootecnico Per La Sicilia, Via Roccazzo 85, 90135 Palermo, Italy

Correction to: *Scientific Reports* 10.1038/s41598-024-73033-3, published online 28 September 2024

The original version of this Article contained errors.

There is a repeated error in the Materials and methods section, where “cDNA” was incorrectly given as “Cdna” and “ddPCR” as “ddpcr”.

In Table 1,

*“Up-regulated genes under category “Male””*

now reads:

*“Up-regulated genes under category “Female””*

*“Down-regulated genes under category “Male”*

now reads:

*“Down-regulated genes under category “Female””*

In the Results section, under the subheading ‘Functional pathways and network analyses’,

“The top significant network mostly incorporated down-regulated genes in the males compared to the females and was associated with molecular transport and cellular organisation (Fig. S2, Supplementary Material 2).”

now reads:

“The top significant network mostly incorporated up-regulated genes in the males compared to the females and was associated with molecular transport and cellular organisation (Fig. S2, Supplementary Material 2).”

In the legend of Figure 5,

“Hypothalamic APOH expression levels (data shown on the natural log-scale) were positively associated with nocturnal activity (i.e. movements/min, data shown on the natural log-scale) only in the migratory birds sampled.”

now reads:

“Hypothalamic APOH expression levels (data shown on the ln-scale) were positively associated with nocturnal activity (i.e. movements/min, data shown on the ln-scale) only in the migratory birds sampled.”

The original Article has been corrected.

