# Supplementary material for: Brain gene expression reveals pathways underlying nocturnal migratory restlessness
Source: Sci Rep. 2024 Sep 28;14:22420. doi: 10.1038/s41598-024-73033-3 (PMC11439032; doi:10.1038/s41598-024-73033-3)
Supplement: Supplementary file 2 — Supplementary Material 2 [file 41598_2024_73033_MOESM2_ESM.docx]

**Supplementary Material**

**Brain gene expression reveals pathways underlying nocturnal migratory restlessness**

Valeria Marasco*^1,2^, Leonida Fusani+^2,3^, Patricia Haubensak^2^, Gianni Pola^4^, Steve Smith^2^+

1 Research Institute of Wildlife Ecology, Department of Interdisciplinary Life Sciences, University of Veterinary Medicine, Vienna, Savoyenstraße 1, 1160, Vienna, Austria

2 Konrad Lorenz Institute of Ethology, Department of Interdisciplinary Life Sciences, University of Veterinary Medicine, Vienna, Savoyenstraße 1a, A-1160, Vienna, Austria

3 Department of Behavioural and Cognitive Biology, University of Vienna, Djerassiplatz 1, 1030, Vienna, Austria.

4 Istituto Sperimentale Zootecnico per la Sicilia, via Roccazzo 85, 90135, Palermo, Italy

*****Corresponding author**:** [valeria.marasco@vetmeduni.ac.at](mailto:valeria.marasco@vetmeduni.ac.at); + joint senior authorship

**Running title**: Pathways regulating migratory movements

## Assessment of food intake and nocturnal activity at sampling

For every individual bird, we measured daily food consumption upon the start of the single housing (10:00–11:00 h) until the following 24 h by weighing the remaining food in the bowl and any food pellet that had been scattered around the cage by the bird. Food intake was calculated as the difference between the mass of the remaining food from the mass of the food provided at the beginning of the experiment. In a subset of birds (n = 12) food intake was measured for two consecutive nights and values were highly repeatable within each individual (r = 0.93, *p* <0.0001 - Lessells and Boag (1987)). For every bird, we also continuously monitored nocturnal locomotor activity during the first two nights since the start of the single housing. Each cage was equipped with an infrared sensor connected with an activity recorder that registered locomotor activity within the cage in 1-min intervals. For each night, we calculated the average activity levels of each bird. Average activity values between the two consecutive nights were highly correlated (Pearson’s test, r = 0.85, *p* < 0.0001; n = 68 birds).

**Droplet Digital PCR (*ddPCR™*)**

We aligned primers using BioEdit Sequence Alignment Editor and assessed their annealing properties and secondary structures with NetPrimer (Premier BioSoft). We used the PrimerQuest Tool by Integrated DNA Technologies (Leuven, Belgium) for performing the probe design. We located the probes in close proximity to either the forward or reverse primer with the annealing temperature being a at least 5°C higher than either primer, and with no guanine base at the 5’ end. Target gene probes were labelled at the 5’ primed end with FAM and reference gene probes with HEX. Both probe types contained BHQ1 as a quencher at the 3’ end.

We first reverse-transcribed RNA to cDNA with MultiScribe™ Reverse Transcriptase (High Capacity cDNA Reverse Transcription Kits, ThermoFisher Scientifc) using random hexamer primers. We designed primer sequences for APOH and LAMP2 from the assembled transcriptome sequences using the NCBI primer design tool. We designed primers and probes for the candidate reference gene, glyceraldehyde-3-phosphate dehydrogenase (GAPDH), from the NCBI reference sequence for *Coturnix japonica* (RefSeq #XM_015873412.2). Details on primer sequences can be found in the Table S3 below.

To assess the optimal concentration for Droplet Digital PCR ddPCR™ we first tested dilution factors for cDNA of 1:1000 and 1:2000. We obtained the best balance of positive to negative droplets across test samples using a cDNA dilution of 1:1000. For the reaction setup, we used 1 µl of the diluted cDNA with the Supermix for Probes (No dUTP) following the manufacturer’s protocol (Bio-Rad Laboratories, California, USA). We portioned the samples into droplets before being transferred into a 96-well plate and sealed with sealing foil. We performed amplification on a GeneAmp® PCR system 9700 (Applied Biosystems, California, USA). We used cycling conditions following the manufacturer’s protocol (ddPCR™ Supermix for Probes (No dUTP), Bio-Rad). We used QX200™ Droplet Reader (Bio-Rad) for data acquisition and performed analyses with the Bio-Rad Droplet Digital™ PCR QuantaSoft software (v. 1.2).

**Cited references**

Boswell T, Hall MR, Goldsmith AR (1993) Annual cycles of migratory fattening, reproduction and moult in European quail (*Coturnix coturnix*). *Journal of Zoology*, **231**, 627-644.

Marasco V, Kaiya H, Pola G, Fusani L (2023) Ghrelin, not corticosterone, is associated with transitioning of phenotypic states in a migratory Galliform. *Frontiers in Endocrinology*, **13**, 1058298.

Marasco V, Sebastiano M, Costantini D, Pola G, Fusani L (2021) Controlled expression of the migratory phenotype affects oxidative status in birds. *Journal of Experimental Biology*, **224**.

**Table S1.** Results of Generalised Linear Mixed Models (GLMMs) with a Gaussian distribution error to assess the effects of the photoperiod manipulation on body mass and subcutaneous fat scores performed in a captive population of common quail to simulate autumn migration followed by a non-migratory life-history stage (see Material and Methods in the main text for full details). Model estimates refer to the factor level indicated in parenthesis; r indicates random factor; in bold significant terms (*p* < 0.05). Sample size for analyses shown in (a) and (b): n = 68; sample size for analyses shown in (c) and (d): n = 35.

|  | (a) Body mass experimental days 0-56 | | | | | (b) Fat scores experimental days 0-56 | | | | |
| --- | --- | --- | --- | --- | --- | --- | --- | --- | --- | --- |
|  | **Estimate** | **SE** | **df** | **t** | ***p*** | **Estimate** | **SE** | **df** | **t** | ***p*** |
| Bird id (r) | 100.410 |  |  |  |  | 4.291 |  |  |  |  |
| Residual | 95.750 |  |  |  |  | 10.403 |  |  |  |  |
| Intercept | 104.474 | 2.155 | 112.242 | 48.485 | <0.0001 | 4.282 | 0.557 | 163.320 | 7.694 | <0.0001 |
| Day | **0.369** | **0.038** | **270.000** | **9.727** | **<0.0001** | **0.103** | **0.012** | **270.000** | **8.256** | **<0.0001** |
| Sex **(**Male**)** | **-6.449** | **3.047** | **112.242** | **-2.116** | **0.037** | -0.488 | 0.787 | 163.320 | -0.620 | 0.536 |
| Time : Sex | 0.035 | 0.054 | 270.000 | 0.662 | 0.509 | 0.007 | 0.018 | 270.000 | 0.369 | 0.713 |
|  |  |  |  |  |  |  |  |  |  |  |
|  | (c) Body mass experimental days 70-105 | | | | | (d) Fat scores experimental days 70-105 | | | | |
|  | **Estimate** | **SE** | **df** | **t** | ***p*** | **Estimate** | **SE** | **df** | **t** | ***p*** |
| Bird id (r) | 184.580 |  |  |  |  | 10.955 |  |  |  |  |
| Residual | 50.410 |  |  |  |  | 4.218 |  |  |  |  |
| Intercept | 153.888 | 6.294 | 166.397 | 24.450 | <0.0001 | 17.413 | 1.755 | 170.917 | 9.924 | <0.0001 |
| Day | **-0.302** | **0.060** | **138.000** | **-4.993** | **<0.0001** | **-0.072** | **0.018** | **138.000** | **-4.117** | **<0.0001** |
| Sex **(**Male**)** | **29.043** | **9.309** | **166.397** | **3.120** | **0.002** | **5.393** | **2.595** | **170.917** | **2.078** | **0.039** |
| Time : Sex | **-0.420** | **0.089** | **138.000** | **-4.699** | **<0.0001** | **-0.071** | **0.026** | **138.000** | **-2.755** | **0.007** |

Slope analysis for significant interaction terms in (c) and (d): the decrease in body mass and fat stores was stronger in males compared to female quails (body mass, male: -0.72 ± 0.07, t = -10.96, *p* < 0.001; body mass, female: -0.30 ± 0.06, t = -4.99, *p* < 0.001; fat scores, male: -0.14 ± 0.02, t = -7.52, *p* < 0.001; fat scores, female: -0.07 ± 0.02, t = -4.12, *p* < 0.001). Data previously published in Marasco *et al.* (2021).

**Table S2.** Results of General Linear models (GLMs) assessing whether levels of (a) food intake or (b) nocturnal activity differed in relation to sampling phase, sex, and their interaction. Model estimates refer to the factor level indicated in parenthesis, in bold significant terms (*p* < 0.05); *non-significant interaction term was removed from the final model. Data previously published in Marasco *et al.* (2023).

1. Food intake

|  | **Estimate** | | **SE** | | **t** | | ***p*** | |  |
| --- | --- | --- | --- | --- | --- | --- | --- | --- | --- |
| Intercept | | 7.720 | | 0.714 | | 10.818 | | <0.0001 | |
| **Phase (Non-Migratory)** | | **-2.125** | | **0.794** | | **-2.676** | | **0.009** | |
| Sex (Male) | | -0.364 | | 0.794 | | -0.459 | | 0.648 | |
| Group: Sex* | |  | |  | |  | | ns | |

1. Nocturnal activity

|  | **Estimate** | | **SE** | | **t** | | ***p*** | |  |
| --- | --- | --- | --- | --- | --- | --- | --- | --- | --- |
| Intercept | | 0.250 | | 0.0501 | | 4.997 | | <0.0001 | |
| **Phase (Non-Migratory)** | | **-0.114** | | **0.0557** | | **-2.044** | | **0.045** | |
| **Sex (Male)** | | **0.125** | | **0.0557** | | **2.246** | | **0.028** | |
| Group: Sex* | |  | |  | |  | | ns | |

**Table S3.** Primer sequences of the reference gene (GAPDH), and the two target genes (APOH and LAMP2).

| **Oligonucleotide name** | **Assay Role** | **Sequence (5'-3')** |
| --- | --- | --- |
| Coturnix_APOH_F | Forward primer | GGA CAA CTT AAG ACC TCT GAG TG |
| Coturnix_APOH_R | Reverse primer | GGG ATA ACA GCA GCA GAG AAA |
| Coturnix_APOH_Probe | Probe | [FAM] AGG CAA ACA TGG GAT GCT CTG AGT [BHQ1] |
| Coturnix_LAMP2_F | Forward primer | AGT ATC TGG ACG AGG ATG TAG G |
| Coturnix_LAMP2_R | Reverse primer | CAC CTT AGG ACC ATG CAC TTA G |
| Coturnix_LAMP2_Probe | Probe | [FAM] ACC ACA GTG CTC TTG TTC AGT CAG T [BHQ1] |
| Ggall_GAPDH_F2 | Forward primer | TTG ACC TGA CCT GCC GTC TG |
| Ggall_GAPDH_R2 | Reverse primer | CAG CAC CCG CAT CAA AGG TG |
| Coturnix_GAPDH_Probe | Probe | [HEX] TCC TGT GAC TTC AAT GGT GAC AGC C [BHQ1] |

**Table S4.** Results of GLMs assessing the effects of sampling phase, sampling time, sex, and their interactions on (a) APOH, or (b) LAMP2 expression levels in the hypothalamus of Common quails. Model estimates refer to the factor level shown in parenthesis. *Non-significant interaction terms (*p* > 0.05) were removed from the final model; significant terms are in bold.

| (a) APOH (ln-transformed) |  |  |  |  |
| --- | --- | --- | --- | --- |
|  | **Estimate** | **SE** | **t** | **p** |
| Intercept | -3.141 | 0.189 | -16.611 | <0.0001 |
| **Sampling phase (migratory)** | 0.696 | 0.256 | 2.715 | 0.009 |
| **Sampling time (night)** | 0.529 | 0.207 | 2.552 | 0.013 |
| **Sex (male)** | 0.451 | 0.208 | 2.171 | 0.034 |
| **Sampling phase : Sampling time** | -0.818 | 0.289 | -2.829 | 0.006 |
| **Sampling phase : Sex** | -0.610 | 0.290 | -2.103 | 0.040 |
| Sampling time : Sex* |  |  |  | ns |
|  |  |  |  |  |
| (b) LAMP2 |  |  |  |  |
|  | Estimate | SE | t | p |
| Intercept | 0.009 | 0.001 | 15.387 | <0.0001 |
| Sampling phase (migratory) | <0.001 | <0.001 | 0.462 | 0.645 |
| Sampling time (night) | <0.001 | <0.001 | 1.838 | 0.071 |
| Sex (male) | <0.001 | <0.001 | 0.861 | 0.393 |
| Sampling phase : Sampling time | -0.002 | 0.001 | -1.842 | 0.070 |
| Sampling phase : Sex* |  |  |  | ns |
| Sampling time : Sex* |  |  |  | ns |

**Table S5.** Results of GLMs assessing the effect of food intake, sex, and nocturnal activity levels on (a) APOH (data ln-transformed) or (b) LAMP2 expression levels in the hypothalamus of Common quails separately by sampling phase (migratory or non-migratory) and sampling time (day or night). Model estimates refer to the factor level in parenthesis; significant terms are in bold.

| **(a) APOH** | |  |  |  |  | |  | | |  | | |  | |  | |  | |  |
| --- | --- | --- | --- | --- | --- | --- | --- | --- | --- | --- | --- | --- | --- | --- | --- | --- | --- | --- | --- |
|  | **Migratory** | **Day** | | | | | |  | | | **Night** | | | | | | | |  |
|  |  | **Estimate** | **SE** | **t** | | **p** | | |  | | | **Estimate** | | **SE** | | **t** | | **p** | |
|  | Intercept | -3.344 | 0.511 | -6.543 | | <0.0001 | | |  | | | -2.862 | | 0.467 | | -6.132 | | <0.0001 | |
|  | Food intake | -0.002 | 0.049 | -0.037 | | 0.971 | | |  | | | -0.017 | | 0.046 | | -0.364 | | 0.722 | |
|  | Sex (male) | 0.201 | 0.347 | 0.579 | | 0.573 | | |  | | | -0.062 | | 0.361 | | -0.171 | | 0.866 | |
|  | Nocturnal Activity | 1.421 | 1.656 | 0.858 | | 0.408 | | |  | | | **1.167** | | **0.387** | | **3.017** | | **0.010** | |
|  |  |  |  |  |  | |  | | |  | | |  | |  | |  | |  |
|  | **Non-migratory** | **Day** | | | | | |  | | | **Night** | | | | | | | |  |
|  |  | **Estimate** | **SE** | **t** | **p** | |  | | | **Estimate** | | | **SE** | | **t** | | **p** | |  |
|  | Intercept | -2.440 | 0.513 | -4.759 | <0.0001 | | | | | -2.733 | | | 0.224 | | -12.197 | | <0.0001 | |  |
|  | Food intake | -0.017 | 0.060 | -0.292 | 0.774 | |  | | | 0.019 | | | 0.027 | | 0.713 | | 0.489 | |  |
|  | Sex (male) | -0.069 | 0.333 | -0.206 | 0.840 | |  | | | -0.266 | | | 0.175 | | -1.524 | | 0.152 | |  |
|  | Nocturnal Activity | 0.131 | 1.182 | 0.111 | 0.913 | |  | | | -0.208 | | | 0.576 | | -0.362 | | 0.723 | |  |
|  |  |  |  |  |  | |  | | |  | | |  | |  | |  | |  |
| **(b) LAMP2** | |  |  |  |  | |  | | |  | | |  | |  | |  | |  |
|  | **Migratory** | **Day** | | | | | |  | | | **Night** | | | | | | | |  |
|  |  | **Estimate** | **SE** | **t** | **p** | |  | | | **Estimate** | | | **SE** | | **t** | | **p** | |  |
|  | Intercept | 0.008 | 0.002 | 5.629 | <0.0001 | |  | | | 0.011 | | | 0.002 | | 6.039 | | <0.0001 | |  |
|  | Food intake | - 0.001 | 0.001 | -0.287 | 0.779 | |  | | | < 0.001 | | | <0.001 | | -0.020 | | 0.985 | |  |
|  | Sex (male) | 0.001 | 0.001 | 1.029 | 0.324 | |  | | | 0.002 | | | 0.001 | | 1.559 | | 0.145 | |  |
|  | Nocturnal Activity | 0.001 | 0.005 | 0.176 | 0.863 | |  | | | -0.003 | | | 0.002 | | -2.079 | | 0.060 | |  |
|  |  |  |  |  |  | |  | | |  | | |  | |  | |  | |  |
|  | **Non-migratory** | **Day** | | | | | |  | | | **Night** | | | | | | | |  |
|  |  | **Estimate** | **SE** | **t** | **p** | |  | | | **Estimate** | | | **SE** | | **t** | | **p** | |  |
|  | Intercept | 0.007 | 0.002 | 4.817 | <0.0001 | | | | | 0.009 | | | 0.001 | | 8.132 | | <0.0001 | |  |
|  | Food intake | <0.001 | <0.001 | 0.111 | 0.913 | |  | | | <0.001 | | | <0.001 | | -0.551 | | 0.591 | |  |
|  | Sex (male) | <0.001 | 0.001 | 0.903 | 0.382 | |  | | | -0.001 | | | 0.001 | | -0.604 | | 0.557 | |  |
|  | Nocturnal Activity | 0.004 | 0.004 | 1.098 | 0.291 | |  | | | <0.001 | | | 0.003 | | -0.098 | | 0.923 | |  |

**
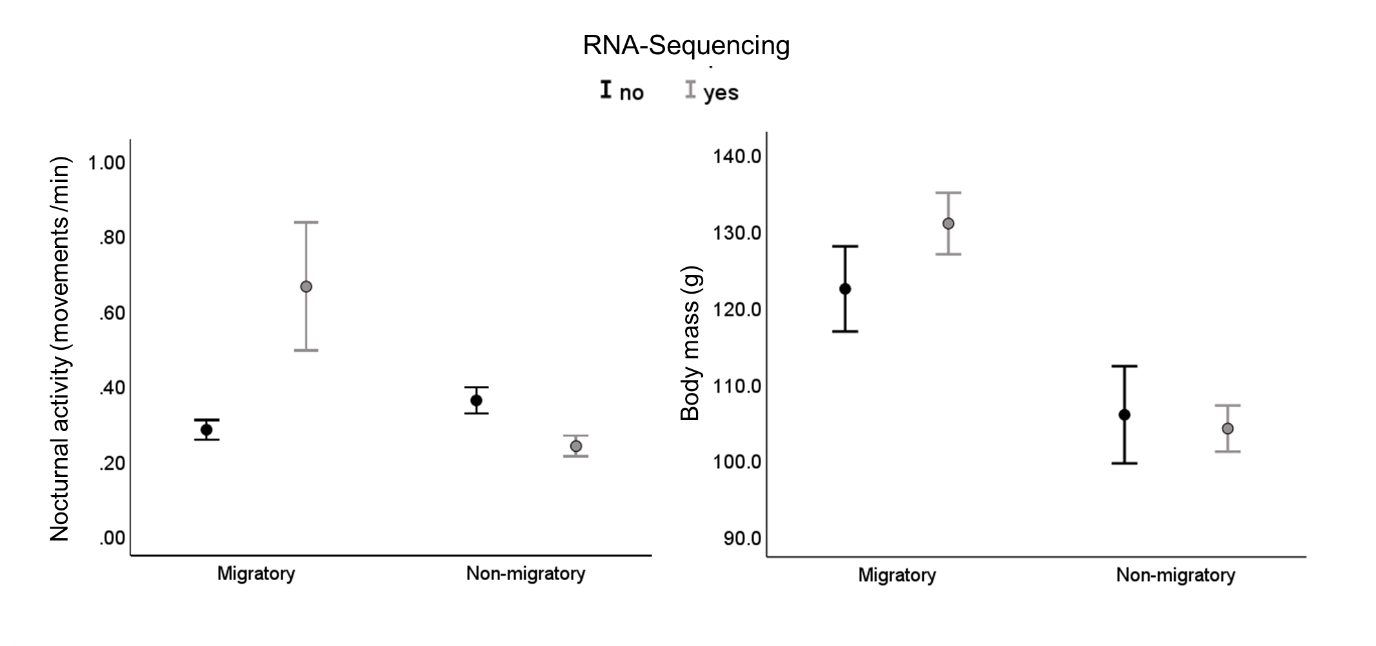
**

**Fig. S1.** Average levels of nocturnal activity (left) and body mass (right) of the common quails sampled at night (Migratory: 8 females and 9 males; Non-Migratory: 10 females and 7 males). Nocturnal activity levels refer to the first part of the night (18:00h-00:00h) during the first night of sampling – see Material and Methods in the main text. The birds showing the largest differences in nocturnal activity levels and body mass were selected for the RNA-Sequencing experiment (Migratory: 6 females and 5 males; Non-migratory: 7 females and 5 males).


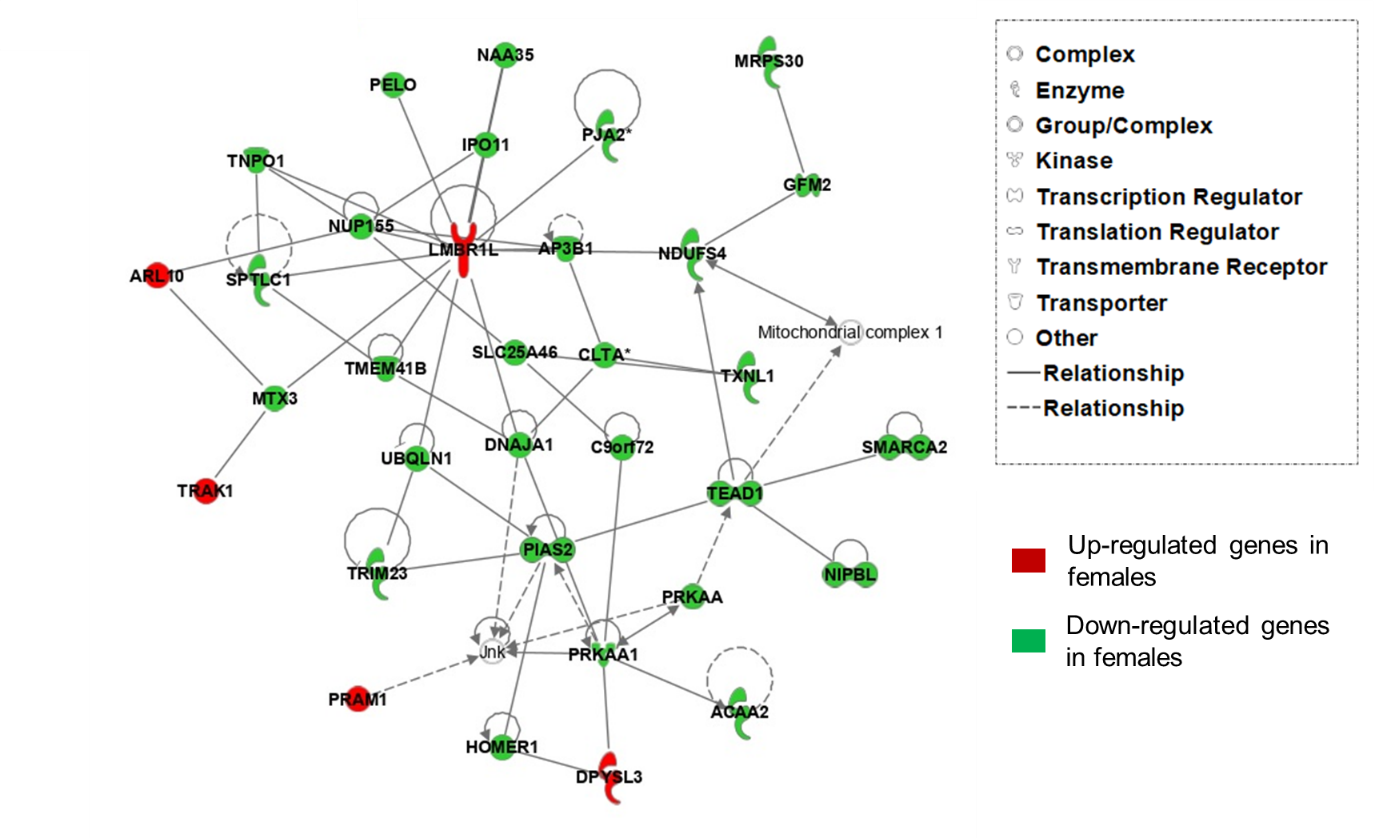


**Fig. S2.** Top significant network generated by Ingenuity Pathway Analysis (IPA) showing the down-regulated focus genes (green) and up-regulated focus genes (red) for the overall effect of sex. Each network is displayed with nodes (i.e. genes) and edges (i.e. biological interactions among nodes); in white, the not user-specific molecules added into the network as a result of interactions with the submitted (i.e. user-specific) genes (green or red). Solid lines connecting distinct molecules indicate direct interactions between the nodes and dashed lines implied indirect interactions.

**
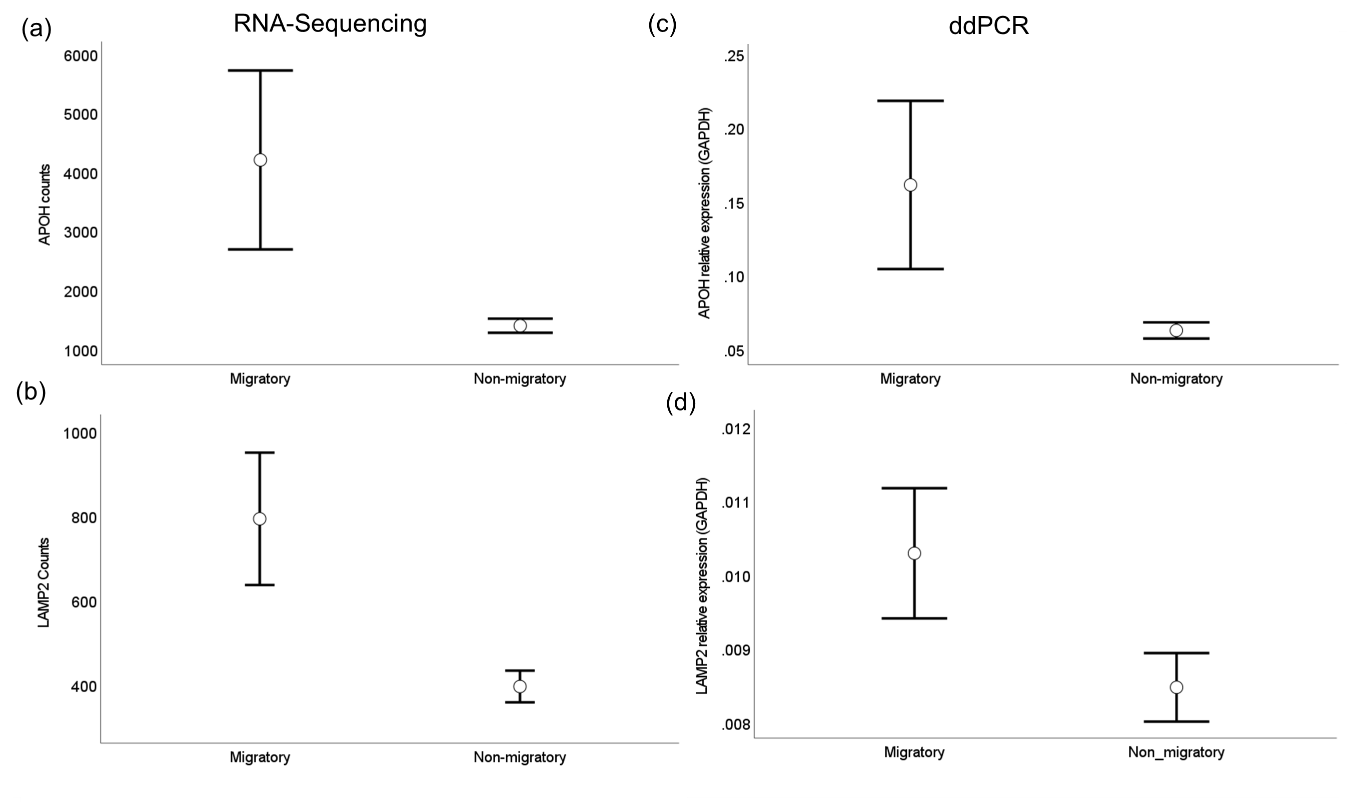
**

**Fig. S3.** Expression values for the genes APOH and LAMP2 from RNA-Sequencing (a, b) and ddPCR (c, d). Data refers to the subset of birds selected for the RNA-Sequencing (n = 23; see Material and Methods in the main text for details).


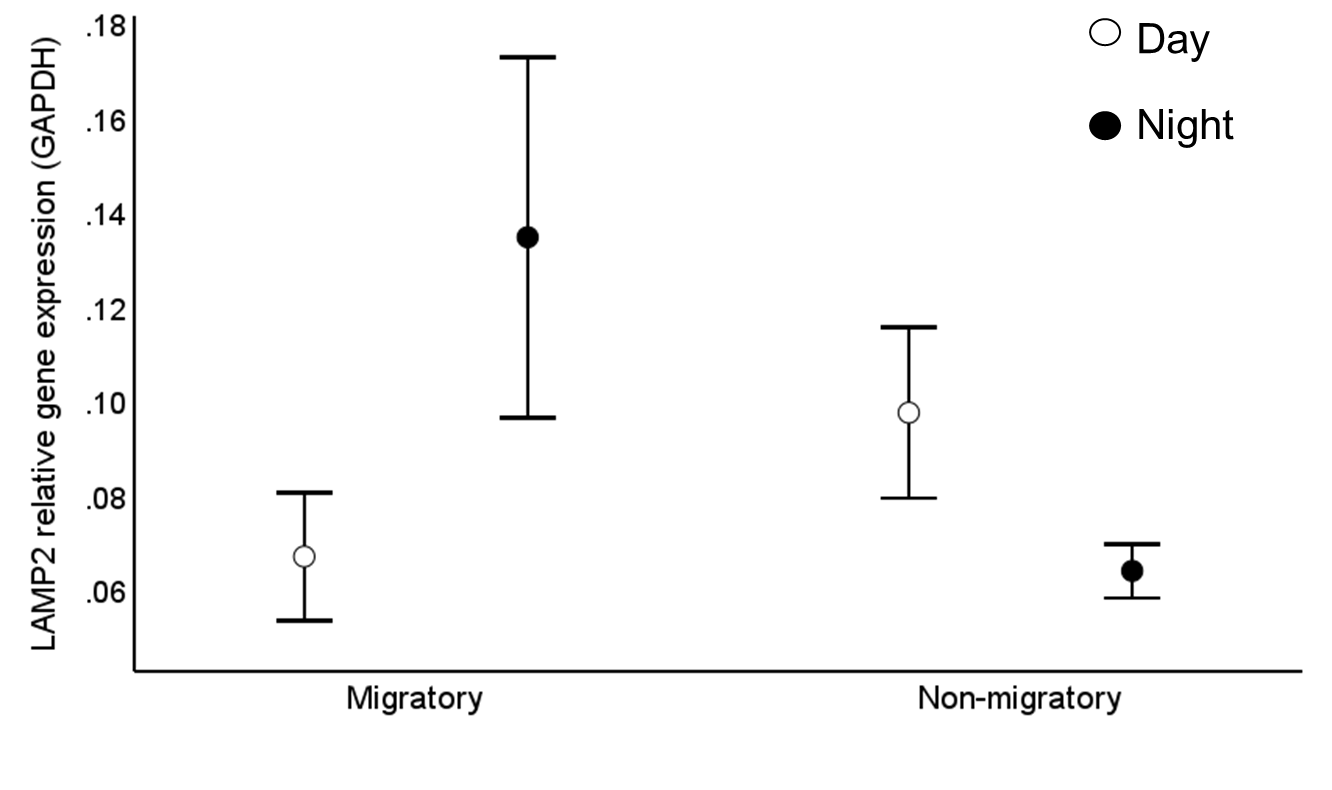


**Fig. S4**. LAMP2 expression levels in brain derived tissues (hypothalamus) of Common quail in relation to sampling phase (migratory or non-migratory) and sampling time (day or night). Data are shown as mean ± sem. See Table S4b for full statistics.


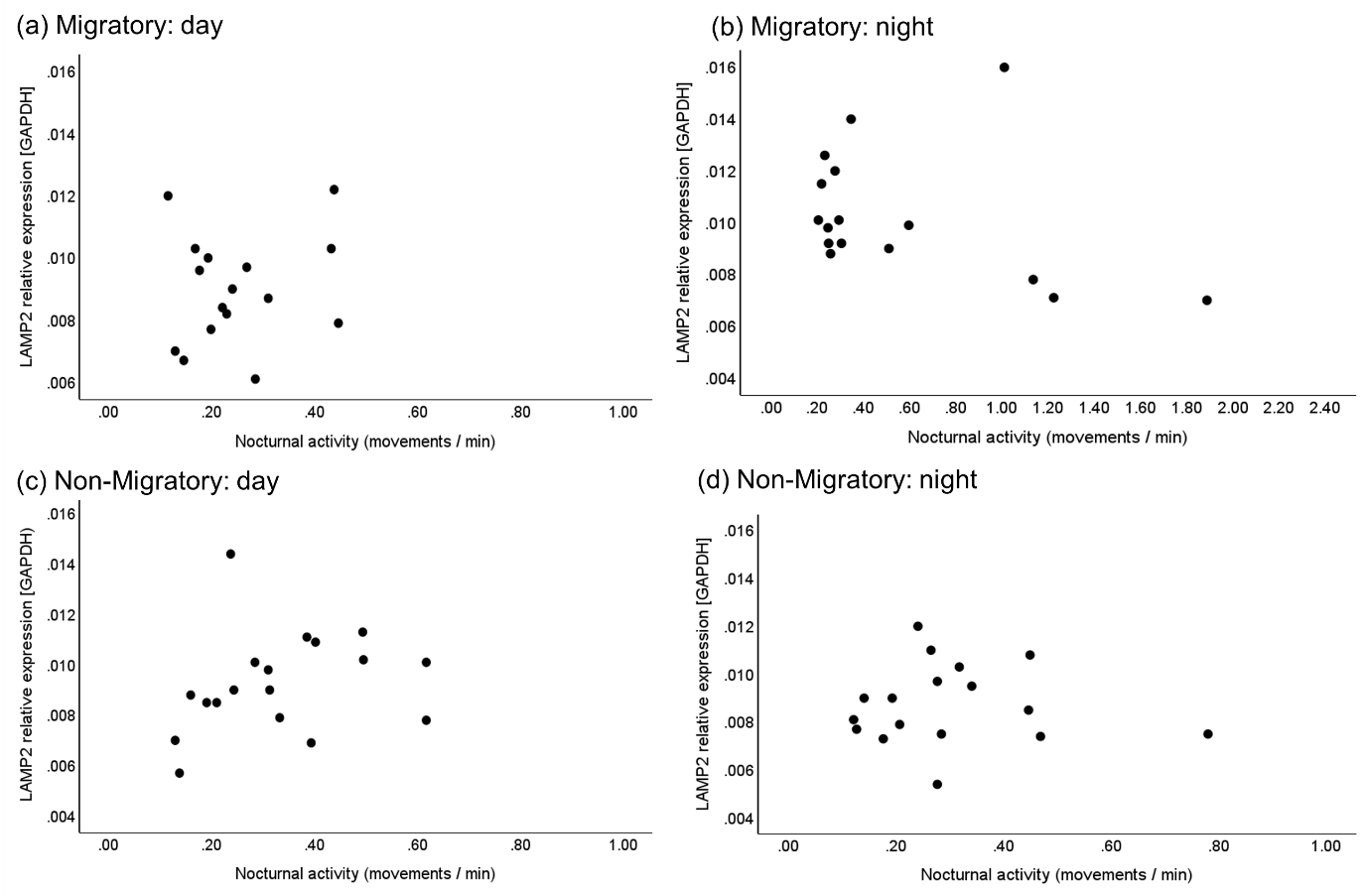


**Fig. S5.** Hypothalamic LAMP2 expression levels were not associated with nocturnal activity levels either in the migratory birds sampled during the day (a) or the night (b), or in the non-migratory birds sampled during the day (c) or night (d). See Table S5 for full statistics.
